# Comparison of the volatile organic compound recovery rates of commercial active samplers for evaluation of indoor air quality in work environments

**DOI:** 10.1007/s11869-017-0465-0

**Published:** 2017-02-18

**Authors:** Yuichi Miyake, Masahiro Tokumura, Qi Wang, Zhiwei Wang, Takashi Amagai

**Affiliations:** Graduate School of Nutritional and Environmental Science, University of Shizuoka, 52-1 Yada, Suruga-ku, Shizuoka, 422-8526 Japan

**Keywords:** Activated carbon, Active sampler, Indoor air, Recovery rate, Work environment

## Abstract

The Industrial Safety and Health Law in Japan established administrative levels for volatile organic compounds (VOCs) in indoor air. In the present study, these 49 VOCs were extracted from the absorbents of commercial active samplers from Sibata Scientific Technology (carbon-bead active sampler), SKC Inc. (Anasorb CSC sorbent tube), and Gastec (bead-shaped activated carbon tube) using carbon disulfide, and the recovery rates were compared. The VOCs were added to the adsorbents at three concentration levels relative to the administrative levels (×0.5, ×1, and ×2). The following mean recovery rates of the 49 VOCs were obtained at the ×0.5, ×1, and ×2 levels: 86, 93, and 92% for the Sibata sampler; 78, 82, and 84% for the SKC sampler; and 94, 93, and 90% for the Gastec sampler. With the Sibata sampler, the recovery rates of 78% (×0.5), 84% (×1), and 90% (×2) of the VOCs measured in this study were adequate (80–120%); the corresponding percentages for the SKC sampler were 67% (×0.5), 69% (×1), and 69% (×2), and those for the Gastec sampler were 92% (×0.5), 86% (×1), and 86% (×2). The effects of the octanol–water partition coefficients and vapor pressures of the VOCs on the recovery rates were investigated. The recovery rates increased with increases in the octanol–water partition coefficient and the vapor pressure and then leveled off. The recovery rates for the *o*-, *m*-, and *p*-cresol isomers were much lower than those obtained for other VOCs at all three concentration levels and with all samplers.

**Electronic supplementary material:**

The online version of this article (doi:10.1007/s11869-017-0465-0) contains supplementary material, which is available to authorized users.

## Introduction

To evaluate indoor air quality, active sampling of indoor air pollutants with an adsorbent tube is used for environmental and occupational applications (Ohura et al. 2009; Gallego et al. 2010; Ramírez et al. 2010; Chin et al. 2013; Jumpponen et al. 2013; Tunsaringkarn et al. 2015; Song et al. 2016). Shinohara et al. (2013) used an active sampler to collect samples to measure 11 volatile organic compounds (VOCs), including toluene, *p*-dichlorobenzene, α-pinene, and aldehydes (formaldehyde and acetaldehyde) in 19 temporary houses in Minami-soma City, Japan, following the Great East Japan earthquake. In another study, nitrogen dioxide concentrations were also measured using an active sampler (Shinohara et al. 2014). In an occupational application, Chen et al. (2014) used an active sampler to collect samples to measure the concentrations of 8 VOCs (e.g., benzene, toluene, and xylenes) in the passenger cabins of 38 taxis in Changsha, China. Their results indicated that VOC concentrations in taxis could be a health risk to passengers and drivers.

Generally, exposure to some VOCs is likely to be higher in occupational settings than in residential indoor and outdoor settings (Jo and Song 2001; Jia et al. 2008; Majumdar et al. 2008; Freberg et al. 2014; Tokumura et al. 2016). Exposure to VOCs from solvent use tends to be high (Leung et al. 2005; Uang et al. 2006; Vitali et al. 2006). Attarchi et al. (2013) reported that workers in a car-manufacturing plant, who were occupationally exposed to VOCs originating from mixed organic solvents (e.g., benzene, toluene, and xylenes), had a high risk of hypertension. In Japan, to protect workers, the Industrial Safety and Health Law established administrative levels for the concentrations of 49 VOCs in indoor work environments, where VOCs are used as solvents (The Japan Association for Working Environment Measurement 2012). The use of active samplers is certified by the Industrial Safety and Health Law in Japan for collection of air samples for VOC analysis.

Nowadays, there are many types of active samplers commercially available (Król et al. 2010; Gallego et al. 2011). Samplers differ in type of adsorbent (e.g., activated carbon, silica gel, and polyurethane foam) and construction (e.g., single layer and double layer), and samplers can be targeted to the physicochemical properties of the VOCs of interest to optimize extraction. Activated carbon is frequently used as an adsorbent for VOCs because it is inexpensive, has a large adsorption capacity, and is adaptable to many types of chemicals. Activated carbons in commercial active samplers can be produced from different precursor materials and differ in their specific surface areas and particle sizes. These characteristics influence adsorption of VOCs and eventually affect the accuracy of the measurement. However, to date, few studies have investigated the effects of these characteristics on accuracy. Borrás et al. (2012) investigated optimization of an active sampler/extraction solvent combination using ORBO-32 activated coconut charcoal (Sigma-Aldrich, St. Louis, MO) and Anasorb CSC coconut charcoal (SKC Ltd., Eighty Four, PA) as the active samplers and hexane and toluene as the extraction solvents. Carbon disulfide in the gas phase was measured to determine the recovery rate, repeatability, reproducibility, and detection limit. According to their results, the Anasorb CSC coconut charcoal sampler in combination with hexane provided adequate sensitivity, good linearity, and a fast and easy protocol for monitoring trace carbon disulfide in air. Abiko (2015) compared the recovery rates of six VOCs (toluene, 1-butanol, acetone, cyclohexanone, ethylene glycol monoethyl ether, and butyl acetate) using eight commercial active samplers to investigate what parameters could directly influence the accuracy of determination. The investigator used activated carbons prepared from coconut shell and petroleum and found that the average particle diameter and the precursor material used to prepare the adsorbent affected the recovery rate. However, not enough samples were analyzed to be able to observe trends in the data, and the recovery rates fluctuated with the type of sampler and VOC. Moreover, the number of VOCs analyzed was limited. Therefore, a more comprehensive study with many kinds of VOCs is required to obtain consistent results.

The aim of this study was to compare the recovery rates obtained with different commercial active samplers for 49 VOCs (Table 1), including isomers, that are included in the Industrial Safety and Health Law in Japan. The commercial active samplers selected were from Sibata, SKC, and Gastec, and the VOCs were added to the absorbents at three concentration levels (0.5, 1, and 2 times the administrative levels) and were extracted using carbon disulfide. The effects of physicochemical properties (octanol–water partition coefficient [log *K*_OW_] and vapor pressure) of the VOCs on the recovery rates were also investigated.

**Table 1:** List of VOCs targeted in this study and their physicochemical properties and administrative levels established by the Industrial Safety and Health Law in Japan

VOC	CAS no.	Molecular weight	Administrative level^a^		Log *K*_OW_ [−]^b^	Vapor pressure (25 °C) [Pa]^b^
			[ppm]	[mg/m^3^]		
Acetone	67–64-1	58.08	500	1190	−0.24	3.32 × 10^4^
Benzene	71–43-2	78.11	1	3.19	1.99	1.16 × 10^4^
1-Butanol	71–36-3	74.12	25	75.8	0.84	1.04 × 10^3^
2-Butanol	78–92-2	74.12	100	303	0.77	2.74 × 10^3^
*n*-Butyl acetate	123–86-4	116.16	150	713	1.85	1.59 × 10^3^
Chlorobenzene	108–90-7	112.56	10	46	2.64	1.24 × 10^3^
Chloroform	67–66-3	119.38	3	14.6	1.52	2.51 × 10^4^
*o*-Cresol	95–48-7	108.14	5	22.1	2.06	3.34 × 10
*m*-Cresol	108–39-4				2.06	2.23 × 10
*p*-Cresol	106–44-5				2.06	1.66 × 10
Cyclohexanol	108–93-0	100.158	25	102	1.64	8.66 × 10
Cyclohexanone	108–94-1	98.15	20	80.3	1.13	5.39 × 10^2^
*p*-Dichlorobenzene	95–50-1	147.01	25	150	3.28	1.29 × 10^2^
1,2-Dichloroethane	107–06-2	98.96	10	40.5	1.83	1.00 × 10^4^
*cis*-1,2-Dichloroethylene	156–59-2	96.95	150	595	1.98	3.38 × 10^4^
Dichloromethane	75–09-2	84.93	50	174	1.34	5.75 × 10^4^
1,2-Dichloropropane	78–87-5	112.99	1	4.62	2.25	6.05 × 10^3^
*N*,*N*-Dimethylformamide	68–12-2	73.09	10	29.9	−0.93	4.65 × 10^2^
1,4-Dioxane	123–91-1	88.11	10	36	−0.32	5.41 × 10^3^
Ethyl acetate	141–78-6	88.11	200	721	0.86	1.31 × 10^4^
Ethyl ether	60–29-7	74.12	400	1210	1.05	7.14 × 10^4^
Ethylene glycol mono-*n*-butyl ether	111–76-2	118.18	25	121	0.57	6.33 × 10
Ethylene glycol monoethyl ether	110–80-5	90.12	5	18.4	−0.42	4.14 × 10^2^
Ethylene glycol monoethyl ether acetate	111–15-9	132.16	5	27	0.59	3.97 × 10^2^
*n*-Hexane	110–54-3	86	40	141	3.29	2.00 × 10^4^
Isobutyl acetate	110–19-0	116.16	150	713	1.77	2.44 × 10^3^
Isobutyl alcohol	78–83-1	74.12	50	152	0.77	1.78 × 10^3^
Isopentyl acetate	123–92-2	130.18	50	266	2.26	7.56 × 10^2^
Isopentyl alcohol	123–51-3	88.15	100	361	1.26	5.12 × 10^2^
Isopropyl acetate	108–21-4	102.1	100	418	1.28	8.11 × 10^3^
Isopropyl alcohol	67–63-0	60.10	200	492	0.28	6.61 × 10^3^
Methyl acetate	79–20-9	74.08	200	606	0.37	7.03 × 10^3^
Methyl *n*-butyl ketone	591–78-6	100.16	5	20.4	1.24	1.81 × 10^3^
1-Methylcyclohexanol	590–67-0	114.19	50	234	2.09	1.36 × 10^2^
2-Methylcyclohexanol	583–59-5				2.05	7.70 × 10
3-Methylcyclohexanol	591–23-1				2.05	6.86 × 10
4-Methylcyclohexanol	589–91-3				2.05	4.58 × 10
2-Methylcyclohexanone	583–60-8	112.17	50	229	1.54	4.01 × 10^2^
3-Methylcyclohexanone	591–24-2				1.54	2.85 × 10^2^
4-Methylcyclohexanone	589–92-4				1.54	2.79 × 10^2^
Methyl ethyl ketone	78–93-3	72.11	200	590	0.26	1.31 × 10^4^
Methyl isobutyl ketone	108–10-1	100.16	20	81.9	1.16	2.90 × 10^3^
*n*-Pentyl acetate	628–63-7	130.19	50	266	2.34	5.55 × 10^2^
*n*-Propyl acetate	109–60-4	102.13	200	835	1.36	4.68 × 10^3^
Styrene	100–42-5	104.15	20	85.2	2.89	6.74 × 10^2^
Tetrachloroethylene	127–18-4	165.83	50	339	2.97	2.37 × 10^3^
Tetrachloromethane	56–23-5	153.82	5	31.5	2.44	1.33 × 10^4^
Tetrahydrofuran	109–99-9	72.11	50	147	0.94	2.31 × 10^4^
Toluene	108–88-3	92.14	20	75.4	2.54	3.16 × 10^3^
1,1,1-Trichloroethane	71–55-6	133.4	200	1090	2.68	1.49 × 10^4^
Trichloroethylene	79–01-6	131.39	10	53.7	2.47	9.66 × 10^3^
*o*-Xylene	95–47-6	106.17	50	217	3.09	9.08 × 10^2^
*m*-Xylene	108–38-3				3.09	8.83 × 10^2^
*p*-Xylene	106–42-3				3.09	9.16 × 10^2^

^a^Established by the Industrial Safety and Health Act in Japan (The Japan Association for Working Environment Measurement 2012)

^b^Obtained from EPI Suite (US EPA 2012)

## Methods

### Chemicals and materials

Standards of 1,1,1-trichloroethane and methyl *n*-butyl ketone were purchased from Tokyo Chemical Industry Co., Ltd. (Tokyo, Japan). Isopropyl alcohol and 1,2-dichloroethane were obtained from Kanto Chemical Co. (Tokyo, Japan) and Dojindo Molecular Technologies, Inc. (Rockville, MD), respectively. All other chemicals were purchased from Wako Pure Chemical Industries, Ltd. (Osaka, Japan). Deuterated toluene (toluene-*d*_8_) was obtained from Cambridge Isotope Laboratories (Tewksbury, MA). Carbon disulfide (Wako Pure Chemical Industries, Ltd.) was used as an extraction solvent. Helium gas (99.999%) was supplied by Taiyo Nippon Sanso Corporation (Tokyo, Japan).

The active samplers purchased for this study were a carbon-bead active sampler (080150–090, Sibata Scientific Technology, Ltd., Saitama, Japan), an Anasorb CSC sorbent tube (SKC 226–01, SKC Inc., Eighty Four, PA, USA), and a bead-shaped activated carbon tube (No. 258, Gastec Co., Ayase, Kanagawa, Japan). The parameters for these commercial active samplers are given in Table 2, and the pictures of them are shown in Fig. S1.

**Table 2:** Parameters for the three commercial active samplers evaluated in this study

Sampler ID	Sibata	SKC	Gastec
Size of sampler	ø 6 mm, length 70 mm	ø 6 mm, length 70 mm	ø 10 mm, length 56 mm
Type of sampler	Double layered	Double layered	Double layered
Separators	FFW	FFW	FFW
Type of adsorbent	Petroleum based	Coconut shell based	Petroleum based
Mass of adsorbent	50/100 mg	50/100 mg	50/100 mg
Size of adsorbent	20/40 mesh	20/40 mesh	20/40 mesh

*F* foam, *W* glass wool

### Determination of recovery rates for VOCs from the adsorbents in the commercial active samplers

Taking into account the air sampling volume (1 L) determined by the analytical method established by the Industrial Safety and Health Act in Japan (The Japan Association for Working Environment Measurement 2012), the amounts of addition of VOCs to the sampler adsorbents for each concentration level were determined. To simplify the addition of VOCs in solvent (carbon disulfide) to the sampler adsorbents, a separate VOC mixed standard solution was prepared for each concentration level so that the volume of this mixed standard solution added to the adsorbent was 4 μL. This resulted in two mixed standards for the ×0.5 level, four mixed standards for the ×1 level, and eight mixed standards for the ×2 level. The VOCs in each of the mixed standards are detailed in Tables S1 to S3. For each mixed standard solution, a 4-μL aliquot was added to the adsorbent in a commercial active sampler. Then, the sampler was stored in a refrigerator overnight. The adsorbent was transferred to a 4-mL vial, and 0.5 mL of toluene-*d*_8_ was added as a syringe spike to correct ionization efficiencies of VOCs during their analysis using gas chromatography–mass spectrometry. The concentration of toluene-*d*_8_ in all samples was 100 μg/mL, except for in the Gastec ×0.5 and ×1 samples, which all had a toluene-*d*_8_ concentration of 56 μg/mL. The adsorbed VOCs were extracted by shaking with 1 mL of carbon disulfide for 60 min. The VOCs in the extract were analyzed by gas chromatography–mass spectrometry using the analytical parameters summarized in Table 3. The recovery rate was calculated by dividing the peak area of the VOC in the extract by the average peak area for a blank solution of the same VOC without an adsorbent, and the resulting value was corrected using the toluene-*d*_8_ peak. The number of each type of sampler used was either five or six.

**Table 3:** Gas chromatography–mass spectrometry conditions for VOC analysis

GC	
Instrument	7890 (Agilent Technologies, Santa Clara, CA)
Column	SUPELCOWAX 10, 60 m × 0.32 mm, 0.5 μm (Sigma-Aldrich)
Injection method	Split (1:450)
Injection volume	1.0 μL
Carrier gas	Helium
Flow rate	1.0 mL/min
Injector temperature	280 °C
Oven temperature program	60 °C (held 5 min) → 230 °C at 6 °C/min (held 2.67 min)
Transfer line temperature	230 °C
MS	
Instrument	Quattro micro (Waters, Milford, MA)
Mode	Selected ion monitoring
Ion source temperature	230 °C
Ionization mode	Electron ionization

## Results and discussion

### Recovery rates for the VOCs from adsorbents in the commercial active samplers

The recovery rates of 49 VOCs added to the adsorbents of the 3 commercial active samplers were evaluated after extraction with carbon disulfide, and the means, standard deviations, and relative standard deviations (RSDs) were calculated (Table 4). The raw data (peak areas of the VOCs in the extracts) are given in Tables S1 to S3. The results are also presented in Fig. S2.

**Table 4:** Summary of the data for the recovery rates of 49 VOCs added to the adsorbents in 3 commercial active samplers at 3 concentration levels and extracted with carbon disulfide

VOC	Multiplier^a^	Sibata			SKC			Gastec		
		Mean (%)	SD (%)	RSD (%)	Mean (%)	SD (%)	RSD (%)	Mean (%)	SD (%)	RSD (%)
Acetone	0.5	101	8.4	8.4	95	17	18	92	13	14
	1	88	5.7	6.5	84	10	12	84	14	16
	2	93	3.9	4.2	74	13	18	75	4.9	6.5
Benzene	0.5	88	5.9	6.7	88	11	12	109	6.9	6.3
	1	97	6.2	6.4	100	10	10	104	5.8	5.5
	2	100	3.5	3.5	106	15	14	102	2.4	2.3
1-Butanol	0.5	69	1.9	2.7	62	11	18	98	7.4	7.6
	1	97	8.1	8.4	71	6.7	9.4	95	8.1	8.5
	2	106	7.1	6.6	78	10	13	92	5.5	6.0
2-Butanol	0.5	93	7.3	7.8	80	10	13	101	8.2	8.2
	1	97	5.0	5.2	85	6.8	8.0	84	9.3	11
	2	87	7.7	8.8	90	13	14	101	7.1	7.0
*n*-Butyl acetate	0.5	112	5.6	5.0	83	7.4	8.9	109	2.1	1.9
	1	105	0.9	0.8	102	2.3	2.3	109	2.1	1.9
	2	102	4.3	4.2	101	2.4	2.3	104	2.5	2.4
Chlorobenzene	0.5	97	3.7	3.8	95	13	14	96	4.2	4.4
	1	103	2.9	2.8	97	2.4	2.4	98	4.0	4.1
	2	96	2.5	2.6	100	3.1	3.1	93	2.4	2.6
Chloroform	0.5	84	7.7	9.2	87	14	16	108	7.3	6.8
	1	100	9.2	9.1	102	11	10	108	10	9.3
	2	99	4.8	4.8	106	10	10	99	6.2	6.3
*o*-Cresol	0.5	6.3	0.4	7.1	1.0	0.5	53	21	3.4	16
	1	31	2.8	9.1	3.6	0.6	16	28	4.1	15
	2	29	3.5	12	8.1	1.0	13	29	3.2	11
*m*-Cresol	0.5	5.9	0.6	10	1.4	0.5	36	12	1.9	16
	1	29	1.3	4.4	3.6	0.8	21	17	3.0	18
	2	24	2.9	12	7.5	0.6	8.4	18	1.7	9.3
*p*-Cresol	0.5	5.4	0.6	12	5.2	3.4	65	11	1.9	18
	1	22	0.9	4.1	3.1	1.4	45	13	1.6	12
	2	20	2.3	12	4.8	0.5	10	13	1.4	10
Cyclohexanol	0.5	81	7.2	8.9	75	13	18	94	2.6	2.7
	1	86	20	23	65	7.4	11	94	4.3	4.6
	2	92	3.8	4.2	77	5.7	7.5	100	9.2	9.2
Cyclohexanone	0.5	105	11	11	76	10	13	91	5.6	6.1
	1	88	5.0	5.7	76	2.8	3.7	94	3.6	3.8
	2	92	7.5	8.1	70	16	23	87	2.5	2.9
*p*-Dichlorobenzene	0.5	83	6.6	7.9	87	19	22	92	7.5	8.2
	1	98	5.4	5.5	86	8.6	10	87	7.3	8.4
	2	91	10.3	11.4	92	6.7	7.3	84	4.5	5.4
1,2-Dichloroethane	0.5	87	7.6	8.8	87	14	16	105	7.3	6.9
	1	103	9.5	9.2	101	10	10	103	7.2	7.1
	2	113	8.7	7.6	100	12	12	99	5.4	5.4
*cis*-1,2-Dichloroethylene	0.5	113	11	10	109	13	12	106	9.4	8.9
	1	108	1.6	1.5	103	8.4	8.2	111	8.8	8.0
	2	108	5.8	5.4	116	9.8	8.5	91	2.6	2.8
Dichloromethane	0.5	72	10	14	84	21	24	108	16	15
	1	92	13	14	102	17	16	104	13	12
	2	97	11	12	102	24	23	96	15	16
1,2-Dichloropropane	0.5	88	3.9	4.4	94	8.7	9.3	105	1.9	1.8
	1	108	7.2	6.7	100	1.9	1.9	105	3.7	3.5
	2	110	3.2	2.9	103	1.4	1.4	100	1.3	1.3
*N*,*N*-Dimethylformamide	0.5	36	2.5	6.9	24	6.0	25	22	6.1	28
	1	73	5.7	7.9	19	3.7	20	30	2.1	7.0
	2	66	3.3	4.9	28	3.2	11	43	2.6	6.2
1,4-Dioxane	0.5	103	10	10	94	6.1	6.5	103	2.5	2.4
	1	112	3.4	3.0	97	3.0	3.1	93	3.7	4.0
	2	109	4.6	4.2	109	4.2	3.9	88	2.2	2.5
Ethyl acetate	0.5	104	5.5	5.3	94	11	12	106	8.4	7.9
	1	98	7.7	7.9	91	7.2	7.9	110	12	10
	2	94	9.3	10	98	10	11	104	11	10
Ethyl ether	0.5	98	8.4	8.6	106	16	15	105	13	12
	1	93	9.2	10	92	12	13	116	19	16
	2	94	16	17	72	22	31	88	8.1	9.2
Ethylene glycol mono-*n*-butyl ether	0.5	70	8.5	12	25	8.6	35	90	5.8	6.5
	1	78	6.2	7.9	25	4.7	18	86	5.9	6.9
	2	87	6.8	7.8	36	1.7	4.6	91	15	16
Ethylene glycol monoethyl ether	0.5	74	2.8	3.7	20	6.6	33	84	5.7	6.8
	1	114	8.6	7.5	75	13	17	59	5.2	8.7
	2	78	8.0	10	11	3.2	30	63	2.1	3.4
Ethylene glycol monoethyl ether acetate	0.5	107	12	11	72	16	23	104	3.0	2.9
	1	70	3.5	5.0	21	2.9	14	84	8.5	10
	2	95	3.1	3.3	91	8.3	9.2	101	4.4	4.3
*n*-Hexane	0.5	95	4.2	4.4	105	9.0	8.6	109	7.3	6.6
	1	109	11	10	97	4.8	5.0	116	11	10
	2	100	8.9	8.9	88	22	25	95	3.6	3.8
Isobutyl acetate	0.5	107	0.7	0.7	88	5.2	5.9	109	2.1	1.9
	1	106	2.1	2.0	98	5.1	5.3	94	4.0	4.3
	2	103	2.0	1.9	100	2.0	2.0	109	3.9	3.6
Isobutyl alcohol	0.5	67	4.2	6.3	83	7.9	9.4	93	5.8	6.3
	1	78	7.3	9.3	83	7.3	8.7	88	9.4	11
	2	95	11	12	92	13	14	96	5.2	5.4
Isopentyl acetate	0.5	101	5.4	5.4	99	12	12	105	4.0	3.8
	1	113	6.6	5.8	101	3.8	3.7	105	3.8	3.7
	2	104	3.8	3.6	98	6.1	6.3	102	4.0	4.0
Isopentyl alcohol	0.5	81	3.6	4.4	70	10	15	97	4.4	4.5
	1	99	7.6	7.7	78	3.0	3.9	97	5.1	5.3
	2	96	7.9	8.2	73	4.7	6.5	88	4.6	5.3
Isopropyl acetate	0.5	112	7.3	6.5	83	12	15	104	5.7	5.4
	1	100	35	35	100	8.6	8.6	115	10	9.0
	2	96	7.3	7.6	91	4.7	5.1	104	5.7	5.5
Isopropyl alcohol	0.5	81	11	14	84	15	18	87	11	13
	1	79	14	18	73	10	14	88	14	16
	2	92	13	15	85	20	24	90	10	11
Methyl acetate	0.5	70	8.0	11	78	18	22	106	17	16
	1	90	34	38	99	13	13	101	13	13
	2	87	9.2	11	81	7.6	9.3	100	11	11
Methyl *n*-butyl ketone	0.5	100	8.1	8.1	90	6.7	7.5	101	5.4	5.3
	1	98	2.9	3.0	99	4.3	4.4	100	4.8	4.8
	2	97	2.4	2.5	98	7.8	8.0	96	2.3	2.4
4-Methylcyclohexanol	0.5	95	10	10	67	21	31	97	5.4	5.6
	1	85	6.8	8.0	61	8.9	15	95	5.7	6.0
	2	87	9.0	10	80	3.8	4.7	91	7.6	8.3
4-Methylcyclohexanone	0.5	98	7.0	7.2	86	17	20	98	6.3	6.5
	1	99	5.6	5.7	83	5.9	7.2	96	6.6	6.9
	2	92	7.0	7.6	88	5.6	6.4	90	2.4	2.7
Methyl ethyl ketone	0.5	66	6.2	9.4	79	15	19	104	10	9.4
	1	94	30	32	92	9.0	10	106	10	10
	2	97	8.6	8.9	87	6.4	7.4	95	6.7	7.0
Methyl isobutyl ketone	0.5	94	2.8	3.0	89	9.3	10	106	2.8	2.7
	1	104	36	34	94	6.8	7.2	115	4.4	3.8
	2	99	2.8	2.9	97	4.8	5.0	109	3.9	3.6
*n*-Pentyl acetate	0.5	100	6.9	6.9	98	13	14	105	5.5	5.2
	1	112	7.2	6.4	98	5.4	5.5	103	4.5	4.3
	2	98	6.9	7.0	96	8.9	9.3	100	6.8	6.8
*n*-Propyl acetate	0.5	99	1.8	1.8	89	9.3	10	105	4.7	4.5
	1	90	17	19	99	7.2	7.3	115	5.9	5.1
	2	97	2.0	2.1	100	5.0	5.0	113	3.1	2.7
Styrene	0.5	81	4.2	5.1	71	12	16	91	5.7	6.2
	1	97	4.6	4.8	74	5.4	7.3	93	4.4	4.7
	2	89	4.6	5.1	87	3.3	3.8	89	2.4	2.7
Tetrachloroethylene	0.5	98	2.8	2.9	95	11	11	100	2.5	2.5
	1	107	4.8	4.4	101	2.1	2.0	101	2.7	2.7
	2	102	0.6	0.6	103	3.2	3.1	96	1.2	1.3
Tetrachloromethane	0.5	97	3.9	4.0	90	12	14	108	5.8	5.4
	1	99	6.5	6.5	103	4.9	4.7	107	6.0	5.6
	2	99	3.4	3.4	104	10	9.4	101	3.2	3.1
Tetrahydrofuran	0.5	82	5.4	6.6	83	13	15	107	6.6	6.1
	1	93	7.3	7.8	96	8.7	9.1	105	7.3	7.0
	2	93	9.1	9.8	86	10	12	89	7.1	8.0
Toluene	0.5	94	2.3	2.4	91	8.8	10	104	3.2	3.1
	1	103	2.6	2.6	101	1.8	1.8	103	2.7	2.7
	2	100	0.5	0.5	103	1.9	1.8	100	1.1	1.1
1,1,1-Trichloroethane	0.5	92	4.0	4.3	90	12	14	107	5.8	5.4
	1	101	29	29	104	8.6	8.3	113	7.3	6.4
	2	99	6.8	6.9	107	11	10	110	4.7	4.3
Trichloroethylene	0.5	99	3.5	3.6	93	10	11	108	4.1	3.8
	1	101	5.6	5.6	104	3.8	3.6	106	4.0	3.8
	2	106	3.3	3.1	109	4.6	4.2	102	1.9	1.8
*o*-Xylene	0.5	100	4.5	4.5	97	14	15	100	3.0	3.0
	1	104	4.7	4.5	97	4.8	5.0	99	5.3	5.3
	2	94	4.2	4.4	101	3.2	3.2	96	3.1	3.2
*m*-Xylene	0.5	110	4.3	3.9	100	14	14	103	4.6	4.5
	1	104	4.4	4.2	100	4.5	4.5	91	4.4	4.9
	2	97	3.5	3.6	103	3.4	3.3	98	3.0	3.1
*p*-Xylene	0.5	103	4.1	4.0	99	14	14	101	4.4	4.3
	1	105	4.5	4.3	99	4.3	4.3	71	3.3	4.6
	2	96	3.8	3.9	103	4.0	3.9	98	2.9	2.9

^a^Factors by which the Industrial Safety and Health Law administrative levels were multiplied

For the Sibata sampler, the recovery rates ranged from 5.4% for *p*-cresol to 113% for *cis*-1,2-dichloroethylene at the ×0.5 level, 22% for *p*-cresol to 114% for ethylene glycol monoethyl ether at the ×1 level, and 20% for *p*-cresol to 113% for 1,2-dichloroethane at the ×2 level. The mean recovery rates for the ×0.5, ×1, and ×2 levels were 86, 93, and 92%, respectively. Satisfaction ratios were calculated as the proportion of VOCs with adequate recovery rates (80–120%). The satisfaction ratios were 78, 84, and 90% for the ×0.5, ×1, and ×2 levels, respectively. Inadequate recovery rates were obtained at some of the concentration levels for 1-butanol (×0.5); *o*-, *m*-, and *p*-cresol (all levels); dichloromethane (×0.5); *N*,*N*-dimethylformamide (all levels); ethylene glycol mono-*n*-butyl ether (×0.5 and ×1); ethylene glycol monoethyl ether (×0.5 and ×2); ethylene glycol monoethyl ether acetate (×1); isobutyl alcohol (×0.5 and ×1); isopropyl alcohol (×1); methyl acetate (×0.5); and methyl ethyl ketone (×0.5). The recovery rates for the cresol isomers were much lower than the recovery rates for any of the other VOCs.

The recovery rates for the SKC sampler ranged from 1.0% for *o*-cresol to 109% for *cis*-1,2-dichloroethylene at the ×0.5 level, 3.1% for *p*-cresol to 104% for trichloroethylene at the ×1 level, and 4.8% for *p*-cresol to 116% for *cis*-1,2-dichloroethylene at the ×2 level. The mean recovery rates for the three levels were 78, 82, and 84%, and the satisfaction ratios were 67, 69, and 69%. Inadequate recovery rates were obtained at some of the concentration levels for acetone (×2); 1-butanol (all levels); *o*-, *m*-, and *p*-cresol (all levels); cyclohexanol (all levels); cyclohexanone (all levels); *N*,*N*-dimethylformamide (all levels); ethyl ether (×2); ethylene glycol mono-*n*-butyl ether (all levels); ethylene glycol monoethyl ether (all levels); ethylene glycol monoethyl ether acetate (×0.5 and ×1); isopentyl alcohol (all levels); isopropyl alcohol (×1); methyl acetate (×0.5); methyl ethyl ketone (×0.5); 4-methylcyclohexanol (×0.5 and ×1); and styrene (×0.5 and ×1). The recovery rates for the cresol isomers, *N*,*N*-dimethylformamide, ethylene glycol mono-*n*-butyl ether, and ethylene glycol monoethyl ether were much lower than the recovery rates for the other VOCs at all the concentration levels.

The recovery rates for the Gastec sampler ranged from 11% for *p*-cresol to 109% for benzene at the ×0.5 level, 13% for *p*-cresol to 116% for *n*-hexane at the ×1 level, and 13% for *p*-cresol to 113% for 1,2-dichloroethane at the ×2 level. The mean recovery rates for the three levels were 94, 93, and 90%, and the satisfaction ratios were 92, 86, and 86%. Inadequate recovery rates were obtained at some of the concentration levels for acetone (×2); *o*-, *m*-, and *p*-cresol (all levels); *N*,*N*-dimethylformamide (all levels); ethylene glycol monoethyl ether (×1 and ×2); and *p*-xylene (×1). The cresols and *N*,*N*-dimethylformamide had much lower recovery rates than the other VOCs at all the concentration levels.

A comparison of the recovery rates among the commercial active samplers showed that the Sibata and Gastec samplers showed good recovery rates. The adsorbents in these samplers are petroleum based. According to an earlier study (Abiko 2015), petroleum-based activated carbons tend to show better recovery rates than coconut shell-based activated carbons. This tendency is in good agreement with our results. Among the VOCs, the cresol isomers (*o*-, *m*-, and *p*-cresol) showed the lowest recovery rates at all concentration levels and with all samplers. The recovery rate of *N*,*N*-dimethylformamide was also much lower than the recovery rates of other VOCs with all samplers except that from Sibata.

The satisfaction ratios for the RSDs (10 or 15%) were 80% (RSD < 10%) and 94% (RSD < 15%) for the Sibata sampler (the petroleum-based adsorbents), 50% (RSD < 10%) and 76% (RSD < 15%) for the SKC sampler (the coconut shell-based adsorbent), and 81% (RSD < 10%) and 92% (RSD < 15%) for the Gastec sampler (the petroleum-based adsorbents). The cresol isomers, dichloromethane, isopropyl alcohol, and methyl acetate likely had higher RSDs at most concentration levels and with most of the samplers. As was the case for the recovery rates, better RSDs were obtained with the petroleum-based adsorbents (Sibata and Gastec) than with the coconut shell-based adsorbent (SKC).

In summary, the satisfaction ratio of adequate recovery rate with adequately low RSD (10 or 15%) were 69% (RSD < 10%) and 78% (RSD < 15%) for the Sibata sampler, 44% (RSD < 10%) and 63% (RSD < 15%) for the SKC sampler, and 76% (RSD < 10%) and 84% (RSD < 15%) for the Gastec sampler.

### Effects of the physicochemical properties of the VOCs on recovery rates

Generally, the recovery rate of a VOC can be affected by its physicochemical properties, and the optimum adsorbent or sampler for a target VOC can be selected on the basis of these properties. In this study, the effects of two physicochemical properties, log *K*_OW_ and vapor pressure, on the recovery rates of the 49 VOCs added at 3 concentration levels to the adsorbents in the 3 commercial active samplers were evaluated after extraction with carbon disulfide.

**Fig. 1:**
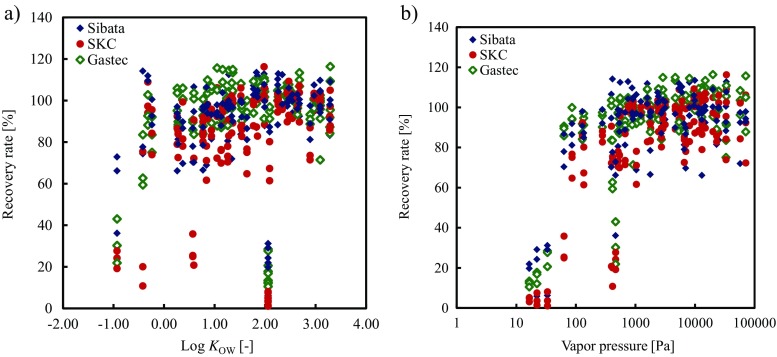
Effects of **a** log *K*_OW_ and **b** vapor pressure on the recovery rates of 49 VOCs added to the adsorbents in 3 commercial active samplers at 3 concentration levels and extracted with carbon disulfide

For log *K*_OW_ (Fig. 1a), the general trend observed was that the recovery rates increased with increases in log *K*_OW_ and then leveled off at around log *K*_OW_ = 0. The solvent used in this study was carbon disulfide, which is non-polar. Therefore, eluting polar VOCs (which generally have relatively low log *K*_OW_ values) from the adsorbents with this solvent was difficult. However, there were some outliers, which were the cresol isomers. Although the cresol isomers all have a log *K*_OW_ of 2.06, their recovery rates ranged from 1 to 31%. With the SKC sampler, ethylene glycol mono-*n*-butyl ether, ethylene glycol monoethyl ether, and ethylene glycol monoethyl ether acetate did not fit the general trend, which suggested that this sampler was incompatible with these specific VOCs.

For the vapor pressure (Fig. 1b), increases in vapor pressure up to 500 Pa led to higher recovery rates. After this point, the recovery rates leveled off. This trend was similar to that observed for log *K*_OW_. Generally, VOCs with lower vapor pressures are more likely to adsorb onto an adsorbent, which could make these VOCs more difficult to desorb than VOCs with higher vapor pressures. Outliers were also found in the vapor pressure data. *N*,*N*-Dimethylformamide, ethylene glycol monoethyl ether, and ethylene glycol monoethyl ether acetate did not follow the general trend. The log *K*_OW_ values of *N*,*N*-dimethylformamide, ethylene glycol monoethyl ether, and ethylene glycol monoethyl ether acetate are −0.93, −0.42, and 0.59, respectively, which are the lowest values among the VOCs measured in this study, except for acetone (−0.24). These results indicate that the recovery rates of these VOCs must be affected more by their log *K*_OW_ values than by their vapor pressures.

On the other hand, polarity of solvent could be a property which could affect the recovery rates of these VOCs. For example, VOCs, which showed low recovery rates (e.g., cresol isomers and *N*,*N*-dimethylformamide), could be expected to be more successfully extracted using polar solvent (e.g., acetone). However, polar solvent would not be adequate for extraction of non-polar VOCs.

## Conclusions

Forty-nine VOCs, for which administrative levels for work environments were established by the Industrial Safety and Health Law in Japan, were added to the adsorbents in three commercial active samplers (Sibata, SKC, and Gastec) at three concentration levels compared to the administrative levels (×0.5, ×1, and ×2) and were extracted using carbon disulfide. The Sibata and Gastec samplers, which are petroleum based, showed good recovery rates and RSDs for the 49 VOCs. Among the VOCs, cresol isomers (*o*-, *m*-, and *p*-cresol) showed the lowest recovery rates at all the concentration levels and with all samplers. With all samplers except for the Sibata sampler, the recovery rate of *N*,*N*-dimethylformamide was much lower than the recovery rates for other VOCs.

An investigation of the effects of two physicochemical properties, log *K*_OW_ and vapor pressure, of the VOCs on the recovery rates showed that the recovery rates increased with increases in log *K*_OW_ and vapor pressure up to a certain point. VOCs with log *K*_OW_ greater than 0 and vapor pressure greater than 500 Pa tended to show good recovery rates.

The comprehensive data of VOC recovery rates could help to select the optimum sampler for evaluation of indoor air quality in work environments.

## Electronic supplementary material

ESM 1 (DOCX 1172 kb)
