# Supplementary material for: Comparison of the volatile organic compound recovery rates of commercial active samplers for evaluation of indoor air quality in work environments
Source: Air Qual Atmos Health. 2017 Feb 18;10(6):737–46. doi: 10.1007/s11869-017-0465-0 (PMC5581818; doi:10.1007/s11869-017-0465-0)
Supplement: Supplementary file 1 — (DOCX 1172 kb) [file 11869_2017_465_MOESM1_ESM.docx]

Supplementary information

**Comparison of Recovery Rates of Commercial Active Samplers for Evaluation of Indoor Air Quality in Work Environment**

Yuichi Miyake, Masahiro Tokumura, Qi Wang, Zhiwei Wang, Takashi Amagai^*^

Graduate School of Nutritional and Environmental Science, University of Shizuoka, Shizuoka, Japan

Corresponding author: Takashi Amagai

Graduate School of Nutritional and Environmental Science, University of Shizuoka, 52-1 Yada, Suruga-ku, Shizuoka 422-8526, Japan

Tel/Fax: +81-54-264-5789

E-mail: amagai@u-shizuoka-ken.ac.jp

Table S1 Raw data (peak areas) for 3 commercial active samplers in concentration of 0.5 × administrative levels.

| 0.5 × Guide line values | | SIBATA | | | | | | | | | | SKC | | | | | | | | | | | GASTEC | | | | | | | | | | |
| --- | --- | --- | --- | --- | --- | --- | --- | --- | --- | --- | --- | --- | --- | --- | --- | --- | --- | --- | --- | --- | --- | --- | --- | --- | --- | --- | --- | --- | --- | --- | --- | --- | --- |
|  |  | Standard | | | | | Sample | | | | | Standard | | | | | Sample | | | | | | Standard | | | | | Sample | | | | | |
|  |  | No. 1 | No. 2 | No. 3 | No. 4 | No. 5 | No. 1 | No. 2 | No. 3 | No. 4 | No. 5 | No. 1 | No. 2 | No. 3 | No. 4 | No. 5 | No. 1 | No. 2 | No. 3 | No. 4 | No. 5 | No. 6 | No. 1 | No. 2 | No. 3 | No. 4 | No. 5 | No. 1 | No. 2 | No. 3 | No. 4 | No. 5 | No. 6 |
| Group 1 | Acetone | 2005 | 1674 | 1936 | 1241 | 1829 | 1802 | 1990 | 1681 | 1569 | 1632 | 59669 | 66183 | 66649 | 74394 | 57593 | 63281 | 47004 | 46578 | 47234 | 48352 | 65436 | 12017 | 11625 | 12816 | 14795 | 12377 | 8861 | 10307 | 10767 | 10227 | 10374 | 9935 |
|  | 2-Butanol | 498 | 564 | 640 | 553 | 584 | 568 | 520 | 485 | 507 | 532 | 11275 | 14041 | 13523 | 15844 | 10849 | 11641 | 7716 | 7749 | 8030 | 7278 | 12615 | 3966 | 3384 | 3328 | 4400 | 3649 | 2942 | 3368 | 3358 | 3520 | 3537 | 2860 |
|  | *n*-Buthyl acetate | 1376 | 1563 | 2091 | 1618 | 1541 | 2216 | 1947 | 1718 | 1602 | 1669 | 31205 | 43445 | 40385 | 62898 | 31257 | 37813 | 24394 | 29467 | 30141 | 19761 | 41475 | 9166 | 7329 | 7769 | 9197 | 7532 | 6437 | 7927 | 7957 | 8914 | 9504 | 6258 |
|  | Cyclohexanone | 276 | 312 | 412 | 277 | 248 | 345 | 300 | 310 | 320 | 302 | 5533 | 8053 | 7529 | 12738 | 6365 | 6187 | 4795 | 5229 | 5397 | 3417 | 6899 | 1107 | 819 | 921 | 1099 | 854 | 581 | 774 | 787 | 883 | 1008 | 601 |
|  | *cis*-1,2-Dichloroethylene | 1585 | 1602 | 1776 | 1478 | 1712 | 1865 | 1846 | 1774 | 1753 | 1856 | 27216 | 37506 | 36892 | 45759 | 31917 | 42335 | 28001 | 29220 | 29815 | 27366 | 48405 | 6415 | 5482 | 5467 | 7211 | 5866 | 4904 | 5785 | 5822 | 6015 | 5883 | 4974 |
|  | 1,4-Dioxane | 80 | 92 | 111 | 83 | 82 | 96 | 89 | 86 | 92 | 93 | 2431 | 3520 | 3308 | 4680 | 2793 | 3348 | 2187 | 2577 | 2606 | 1991 | 3871 | 450 | 329 | 353 | 449 | 344 | 280 | 365 | 359 | 407 | 393 | 274 |
|  | Ethyl acetate | 647 | 710 | 781 | 657 | 724 | 803 | 757 | 680 | 679 | 708 | 11462 | 15380 | 14227 | 17058 | 11834 | 14801 | 9297 | 9878 | 10064 | 8978 | 15944 | 4291 | 3524 | 3512 | 4604 | 3754 | 3177 | 3759 | 3742 | 3972 | 3849 | 3186 |
|  | Ethyl ether | 1629 | 1285 | 1540 | 931 | 1194 | 1423 | 1550 | 1157 | 1216 | 1084 | 30727 | 37747 | 37035 | 43335 | 30285 | 42704 | 28729 | 27063 | 28884 | 26100 | 45023 | 10195 | 9611 | 9404 | 12299 | 10070 | 8306 | 9537 | 10003 | 9654 | 9666 | 8847 |
|  | Ethylene glycol mono-*n*-butyl ether | 276 | 312 | 322 | 287 | 253 | 198 | 258 | 182 | 188 | 189 | 7086 | 9857 | 8141 | 14936 | 6697 | 2377 | 2386 | 1699 | 2176 | 878 | 2585 | 997 | 831 | 956 | 1142 | 881 | 624 | 829 | 755 | 793 | 962 | 568 |
|  | Ethylene glycol monoethyl ether | 59 | 73 | 81 | 68 | 59 | 57 | 54 | 46 | 48 | 47 | 1841 | 2442 | 2050 | 3143 | 1805 | 595 | 417 | 272 | 331 | 187 | 616 | 504 | 444 | 435 | 547 | 484 | 304 | 388 | 381 | 379 | 384 | 274 |
|  | Ethylene glycol monoethyl ether acetate | 68 | 79 | 108 | 76 | 63 | 90 | 79 | 75 | 85 | 89 | 2036 | 2723 | 2395 | 3771 | 1915 | 1905 | 1611 | 1387 | 1807 | 930 | 1985 | 361 | 307 | 354 | 397 | 316 | 256 | 328 | 314 | 352 | 391 | 250 |
|  | *n*-Hexane | 54 | 49 | 54 | 41 | 41 | 51 | 50 | 38 | 44 | 45 | 2061 | 2954 | 2906 | 3869 | 2395 | 3255 | 2060 | 2296 | 2377 | 1965 | 3744 | 545 | 465 | 430 | 578 | 444 | 405 | 488 | 488 | 532 | 494 | 398 |
|  | Isobutyl acetate | 3060 | 3353 | 4153 | 3284 | 3227 | 4330 | 3950 | 3186 | 3378 | 3459 | 64983 | 94185 | 87078 | 131387 | 70362 | 85181 | 52957 | 66925 | 66859 | 47167 | 97692 | 23236 | 18401 | 18849 | 22726 | 18786 | 16316 | 19881 | 19759 | 22311 | 22824 | 15795 |
|  | Isobutyl alcohol | 108 | 114 | 108 | 92 | 87 | 71 | 74 | 63 | 63 | 65 | 9379 | 12895 | 12494 | 15302 | 10320 | 10730 | 7151 | 7650 | 7991 | 6807 | 12646 | 1029 | 873 | 794 | 1115 | 930 | 659 | 803 | 818 | 826 | 839 | 640 |
|  | Isopropyl alcohol | 3474 | 3163 | 3405 | 2777 | 3335 | 2618 | 2546 | 2757 | 2395 | 2648 | 91661 | 113181 | 116669 | 130158 | 104989 | 98646 | 73358 | 62623 | 73583 | 68628 | 105298 | 16601 | 16235 | 18216 | 21906 | 18116 | 11958 | 14060 | 15083 | 13664 | 14418 | 13134 |
|  | 4-Methylcyclohexanol | 544 | 897 | 346 | 486 | 491 | 512 | 350 | 490 | 373 | 336 | 7111 | 4961 | 5458 | 7081 | 5646 | 3251 | 4659 | 5441 | 3894 | 6937 | 6343 | 2570 | 2046 | 2404 | 2689 | 2180 | 2187 | 2405 | 2112 | 2136 | 1627 | 1429 |
|  | toluene-*d*_8_ | 2080 | 2143 | 2636 | 2023 | 1954 | 2545 | 2352 | 1894 | 1982 | 2052 | 32851 | 53526 | 51304 | 84673 | 44764 | 53367 | 34016 | 46753 | 45306 | 34118 | 69933 | 8957 | 6918 | 7430 | 8468 | 6883 | 9563 | 12096 | 11799 | 13559 | 13821 | 9208 |
| Group 2 | Benzene | 20 | 19 | 21 | 35 | 15 | 19 | 19 | 18 | 20 | 21 | 808 | 770 | 768 | 793 | 715 | 769 | 849 | 609 | 894 | 914 | 922 | 179 | 166 | 222 | 209 | 193 | 249 | 197 | 222 | 185 | 220 | 194 |
|  | 1-Butanol | 273 | 275 | 276 | 471 | 193 | 228 | 191 | 207 | 197 | 213 | 5580 | 4772 | 4675 | 5229 | 4627 | 3508 | 3587 | 2606 | 4529 | 4295 | 3748 | 1649 | 1639 | 2174 | 1927 | 1950 | 2016 | 1709 | 1887 | 1741 | 1832 | 1635 |
|  | Chlorobenzene | 876 | 930 | 871 | 1639 | 523 | 1036 | 839 | 992 | 911 | 951 | 8607 | 9013 | 8848 | 9703 | 7193 | 9609 | 8925 | 6449 | 10398 | 12029 | 13077 | 2426 | 1898 | 2699 | 2505 | 2396 | 2618 | 2194 | 2536 | 2004 | 2184 | 2159 |
|  | Chloroform | 79 | 82 | 84 | 135 | 57 | 84 | 74 | 74 | 67 | 70 | 2834 | 2465 | 2352 | 2713 | 2453 | 2586 | 2541 | 1978 | 3198 | 3265 | 2705 | 370 | 385 | 495 | 441 | 415 | 541 | 417 | 480 | 435 | 461 | 384 |
|  | *o*-Cresol | 221 | 221 | 236 | 469 | 118 | 19 | 13 | 15 | 17 | 17 | 2870 | 3488 | 3514 | 3170 | 2519 | 30 | 14 | 30 | 67 | 29 | 71 | 799 | 571 | 853 | 792 | 753 | 165 | 158 | 153 | 172 | 128 | 142 |
|  | *p*-Cresol | 49 | 48 | 52 | 103 | 32 | 4 | 3 | 3 | 3 | 3 | 894 | 1113 | 1095 | 883 | 788 | 43 |  | 55 | 115 | 67 | 87 | 228 | 138 | 223 | 204 | 205 | 21 | 24 | 26 | 20 | 15 | 19 |
|  | *m*-Cresol | 279 | 275 | 302 | 605 | 161 | 22 | 16 | 22 | 19 | 19 | 4354 | 5225 | 5683 | 4716 | 4053 | 50 | 63 | 75 | 123 | 58 | 123 | 960 | 666 | 1050 | 961 | 913 | 121 | 109 | 114 | 123 | 87 | 101 |
|  | Cyclohexanol | 382 | 397 | 370 | 688 | 192 | 368 | 289 | 375 | 319 | 313 | 6675 | 7194 | 7089 | 7349 | 5882 | 5784 | 5340 | 3907 | 7446 | 6975 | 8318 | 1643 | 1263 | 1775 | 1734 | 1626 | 1781 | 1390 | 1683 | 1363 | 1463 | 1380 |
|  | *o*-Dichlorobenzene | 1233 | 1245 | 1234 | 2161 | 866 | 1260 | 1006 | 1252 | 1078 | 1071 | 7601 | 8853 | 8702 | 8888 | 6319 | 7296 | 6747 | 5204 | 10805 | 10144 | 11343 | 4542 | 3379 | 4664 | 4652 | 4065 | 4408 | 3957 | 4106 | 3714 | 3485 | 3587 |
|  | 1,2-Dichloroethane | 54 | 53 | 49 | 82 | 33 | 54 | 47 | 41 | 45 | 52 | 2109 | 1917 | 1760 | 2104 | 1774 | 2184 | 1908 | 1421 | 2363 | 2292 | 2083 | 301 | 313 | 417 | 365 | 339 | 433 | 338 | 393 | 340 | 380 | 299 |
|  | Dichloromethane | 358 | 328 | 391 | 520 | 274 | 306 | 298 | 239 | 252 | 260 | 9169 | 7258 | 7042 | 8486 | 7965 | 7437 | 7283 | 6227 | 11148 | 9483 | 7063 | 1722 | 1813 | 2214 | 1776 | 1751 | 2099 | 2087 | 1869 | 2042 | 2114 | 1665 |
|  | 1,2-Dichloropropane | 120 | 121 | 110 | 225 | 76 | 133 | 107 | 114 | 109 | 119 | 6507 | 6641 | 6612 | 7298 | 5556 | 7799 | 6865 | 5383 | 7311 | 8439 | 9349 | 933 | 775 | 1074 | 1062 | 926 | 1219 | 918 | 1134 | 866 | 963 | 892 |
|  | *N,N*-Dimethylformamide | 95 | 85 | 96 | 169 | 56 | 38 | 37 | 34 | 36 | 38 | 2470 | 2816 | 3048 | 2663 | 2164 | 649 | 628 | 659 | 1104 | 697 | 883 | 762 | 587 | 969 | 892 | 852 | 167 | 236 | 152 | 160 | 136 | 191 |
|  | Isopenthyl alcohol | 851 | 883 | 840 | 1390 | 523 | 804 | 658 | 769 | 698 | 725 | 13859 | 13217 | 12990 | 14289 | 11671 | 11441 | 10547 | 7600 | 13027 | 12257 | 13014 | 4527 | 4207 | 5422 | 5065 | 4960 | 5410 | 4294 | 5185 | 4334 | 4735 | 4132 |
|  | Isopentyl acetate | 1271 | 1326 | 1269 | 2323 | 739 | 1583 | 1270 | 1492 | 1351 | 1379 | 22041 | 22656 | 23013 | 24878 | 18591 | 25890 | 23595 | 17923 | 27499 | 31879 | 34409 | 7860 | 6204 | 8385 | 8123 | 7724 | 9263 | 7591 | 8752 | 7168 | 7856 | 7275 |
|  | Isopropyl acetate | 464 | 501 | 501 | 861 | 407 | 658 | 571 | 570 | 594 | 720 | 13029 | 11429 | 11237 | 12478 | 10951 | 11847 | 11314 | 8707 | 13798 | 13359 | 12209 | 17567 | 17928 | 22504 | 21050 | 18921 | 23863 | 19099 | 21694 | 18750 | 20628 | 17736 |
|  | Methyl acetate | 448 | 371 | 480 | 515 | 377 | 279 | 293 | 310 | 285 | 375 | 12843 | 10415 | 9593 | 12234 | 11452 | 9424 | 9816 | 8459 | 14302 | 12087 | 9611 | 3310 | 3438 | 4215 | 3393 | 3322 | 3928 | 3907 | 3435 | 3817 | 3973 | 3065 |
|  | Methyl *n*-buthyl ketone | 10 | 10 | 11 | 17 | 6 | 11 | 11 | 10 | 11 | 12 | 348 | 439 | 390 | 368 | 273 | 410 | 345 | 303 | 403 | 434 | 489 | 44 | 36 | 48 | 45 | 44 | 52 | 42 | 47 | 38 | 43 | 42 |
|  | Methyl ethyl ketone | 1196 | 1108 | 1186 | 1594 | 870 | 920 | 759 | 691 | 721 | 898 | 21234 | 17836 | 16856 | 20192 | 17668 | 17101 | 16472 | 13175 | 22690 | 20716 | 17164 | 5776 | 5968 | 7190 | 6265 | 6083 | 7179 | 6244 | 6501 | 6249 | 6695 | 5618 |
|  | Methyl isobutyl ketone | 69 | 77 | 73 | 136 | 50 | 84 | 67 | 79 | 72 | 82 | 2165 | 2345 | 2022 | 2297 | 1764 | 2315 | 2191 | 1629 | 2407 | 2488 | 2812 | 340 | 308 | 400 | 383 | 357 | 453 | 348 | 417 | 333 | 368 | 339 |
|  | 4-Methylcyclohexanone | 541 | 578 | 567 | 1084 | 314 | 672 | 525 | 670 | 598 | 588 | 6106 | 6923 | 6945 | 7158 | 5322 | 6206 | 5451 | 4134 | 8279 | 8164 | 8900 | 2079 | 1565 | 2177 | 2134 | 2027 | 2175 | 1856 | 2047 | 1793 | 1861 | 1840 |
|  | *n*-Pentyl acetate | 1088 | 1130 | 1105 | 1999 | 596 | 1348 | 1076 | 1279 | 1147 | 1133 | 18452 | 20054 | 19813 | 21175 | 15532 | 21392 | 19630 | 15115 | 24498 | 27160 | 29346 | 6889 | 5349 | 6963 | 6803 | 6562 | 7724 | 6543 | 7318 | 6251 | 6479 | 6276 |
|  | *n*-Propyl acetate | 3263 | 3373 | 3220 | 5506 | 2487 | 3874 | 3275 | 3486 | 3457 | 3849 | 54685 | 51097 | 49189 | 54994 | 45414 | 55318 | 51870 | 40621 | 60028 | 60721 | 63407 | 18481 | 17524 | 22288 | 21141 | 19425 | 24051 | 19226 | 22267 | 18795 | 20680 | 18457 |
|  | Styrene | 1619 | 1681 | 1619 | 2906 | 950 | 1568 | 1288 | 1546 | 1384 | 1423 | 18217 | 19812 | 19593 | 21165 | 15439 | 15004 | 14188 | 10326 | 17684 | 20720 | 21342 | 5032 | 3822 | 5278 | 5007 | 4809 | 4913 | 4211 | 4615 | 3955 | 4093 | 4133 |
|  | Tetrachloroethylene | 1183 | 1278 | 1193 | 2292 | 784 | 1487 | 1195 | 1332 | 1281 | 1353 | 7444 | 7415 | 7477 | 8222 | 6087 | 8513 | 7772 | 5800 | 8332 | 9944 | 10854 | 3850 | 3171 | 4213 | 4188 | 3830 | 4594 | 3542 | 4284 | 3422 | 3771 | 3471 |
|  | Tetrachloromethane | 1642 | 1690 | 1682 | 2822 | 1310 | 1908 | 1675 | 1690 | 1742 | 1979 | 21165 | 19120 | 18705 | 20970 | 17937 | 21888 | 19949 | 15416 | 24021 | 24055 | 22412 | 4415 | 4436 | 5506 | 5168 | 4671 | 6040 | 4882 | 5587 | 4796 | 5308 | 4658 |
|  | Tetrahydrofuran | 216 | 212 | 223 | 362 | 160 | 213 | 179 | 171 | 189 | 216 | 5173 | 5097 | 4417 | 4978 | 4336 | 5103 | 4399 | 3688 | 5650 | 5224 | 4750 | 1635 | 1675 | 2047 | 1908 | 1715 | 2202 | 1827 | 2015 | 1750 | 1952 | 1684 |
|  | Toluene | 730 | 780 | 729 | 1404 | 472 | 861 | 674 | 790 | 763 | 829 | 18160 | 18377 | 18247 | 19787 | 15151 | 20492 | 18604 | 14148 | 19791 | 22106 | 24996 | 3380 | 2821 | 3753 | 3704 | 3313 | 4130 | 3260 | 3873 | 3122 | 3444 | 3197 |
|  | 1,1,1-Trichloroethane | 11711 | 11859 | 11760 | 18492 | 9360 | 12736 | 11028 | 11034 | 11302 | 12958 | 217007 | 193740 | 190381 | 210124 | 182061 | 222252 | 201082 | 158099 | 246123 | 242571 | 224846 | 35189 | 35462 | 43878 | 40969 | 37023 | 47871 | 38513 | 43519 | 37678 | 41464 | 36201 |
|  | Trichloroethylene | 164 | 166 | 158 | 331 | 112 | 206 | 170 | 176 | 180 | 205 | 2520 | 2356 | 2302 | 2625 | 2101 | 2831 | 2541 | 1887 | 2782 | 2986 | 3104 | 774 | 740 | 975 | 922 | 828 | 1090 | 831 | 1009 | 820 | 918 | 790 |
|  | *o*-Xylene | 2507 | 2661 | 2575 | 4632 | 1581 | 3030 | 2508 | 2979 | 2748 | 2785 | 30199 | 32306 | 32534 | 34611 | 25424 | 34657 | 31352 | 23718 | 37089 | 46690 | 47249 | 7642 | 5757 | 7758 | 7706 | 7125 | 8372 | 6786 | 7983 | 6342 | 6917 | 6571 |
|  | *m*-Xylene | 2208 | 2339 | 2271 | 4255 | 1466 | 2967 | 2473 | 2938 | 2729 | 2799 | 28121 | 29817 | 29779 | 32759 | 23538 | 33116 | 30412 | 23368 | 34862 | 44443 | 45654 | 8325 | 6404 | 8717 | 8549 | 7932 | 9403 | 7833 | 8842 | 7220 | 8045 | 7485 |
|  | *p*-Xylene | 1102 | 1187 | 1135 | 2132 | 674 | 1430 | 1139 | 1354 | 1250 | 1297 | 14416 | 15451 | 15398 | 16709 | 12037 | 16892 | 15437 | 12017 | 17758 | 22829 | 23287 | 4277 | 3260 | 4445 | 4331 | 4017 | 4729 | 3983 | 4477 | 3606 | 3946 | 3767 |
|  | toluene-*d*_8_ | 1653 | 1746 | 1603 | 3062 | 1144 | 2000 | 1671 | 1858 | 1813 | 1971 | 35076 | 35720 | 35007 | 38585 | 29186 | 40883 | 37759 | 37190 | 40221 | 45730 | 51526 | 7277 | 6197 | 8043 | 7797 | 7042 | 14938 | 11520 | 14573 | 11019 | 11892 | 11120 |

Table S2 Raw data (peak areas) for 3 commercial active samplers in concentration of 1 × administrative levels.

| 1 × Guide line values | | SIBATA | | | | | | | | | | SKC | | | | | | | | | | | GASTEC | | | | | | | | | | |
| --- | --- | --- | --- | --- | --- | --- | --- | --- | --- | --- | --- | --- | --- | --- | --- | --- | --- | --- | --- | --- | --- | --- | --- | --- | --- | --- | --- | --- | --- | --- | --- | --- | --- |
|  |  | Standard | | | | | Sample | | | | | Standard | | | | | Sample | | | | | | Standard | | | | | Sample | | | | | |
|  |  | No. 1 | No. 2 | No. 3 | No. 4 | No. 5 | No. 1 | No. 2 | No. 3 | No. 4 | No. 5 | No. 1 | No. 2 | No. 3 | No. 4 | No. 5 | No. 1 | No. 2 | No. 3 | No. 4 | No. 5 | No. 6 | No. 1 | No. 2 | No. 3 | No. 4 | No. 5 | No. 1 | No. 2 | No. 3 | No. 4 | No. 5 | No. 6 |
| Group 1 | Acetone | 296050 | 280928 | 320567 | 269613 | 268454 | 223531 | 216480 | 226275 | 223805 | 208178 | 41243 | 46362 | 45600 | 47367 | 49498 | 51269 | 41278 | 48239 | 43248 | 41614 | 36239 | 15673 | 13401 | 17449 | 12303 | 9769 | 9093 | 8158 | 9357 | 8319 | 6370 | 6085 |
|  | 2-Butanol | 164687 | 159032 | 145579 | 141214 | 137509 | 129513 | 119342 | 138414 | 121628 | 120737 | 10820 | 9545 | 9221 | 8890 | 9236 | 10805 | 8496 | 9550 | 9320 | 8396 | 8240 | 3137 | 3335 | 3883 | 2180 | 2214 | 2089 | 1640 | 2095 | 1725 | 1363 | 1403 |
|  | *cis*-1,2-Dichloroethylene | 513167 | 524967 | 535920 | 509272 | 478183 | 485003 | 432742 | 513687 | 485677 | 480181 | 39439 | 33032 | 34643 | 32534 | 34174 | 47243 | 38007 | 41505 | 40850 | 36838 | 36749 | 7294 | 7794 | 9271 | 5754 | 5814 | 6485 | 4976 | 6491 | 5562 | 4716 | 4815 |
|  | 1,4-Dioxane | 85050 | 87340 | 84041 | 82742 | 79139 | 78657 | 72941 | 90884 | 80798 | 83477 | 3497 | 2498 | 2532 | 2255 | 2484 | 3465 | 2601 | 3052 | 3196 | 2434 | 2798 | 414 | 472 | 639 | 279 | 357 | 378 | 227 | 349 | 260 | 227 | 244 |
|  | Ethylene glycol monoethyl ether | 43222 | 40083 | 48766 | 39755 | 57645 | 42710 | 42050 | 48653 | 52129 | 42103 | 1756 | 1434 | 1262 | 1224 | 1426 | 1683 | 1282 | 1171 | 1235 | 844 | 1090 | 282 | 314 | 387 | 217 | 195 | 160 | 101 | 142 | 112 | 85 | 98 |
|  | Isobutyl acetate | 915013 | 884077 | 889994 | 830368 | 833833 | 783716 | 715295 | 892166 | 802580 | 800576 | 75571 | 53100 | 52491 | 44950 | 50605 | 73437 | 55757 | 64338 | 61297 | 51999 | 61196 | 16219 | 17949 | 23387 | 11177 | 12964 | 14014 | 9050 | 13351 | 9962 | 8800 | 9245 |
|  | Isobutyl alcohol | 3799 | 3496 | 3064 | 2982 | 2599 | 2453 | 1985 | 2391 | 1993 | 2041 | 7948 | 6743 | 6690 | 6340 | 6757 | 7561 | 6258 | 6650 | 6658 | 5860 | 5819 | 1026 | 1132 | 1364 | 741 | 765 | 732 | 564 | 786 | 621 | 483 | 497 |
|  | Isopropyl alcohol | 166378 | 153286 | 155157 | 132718 | 125045 | 106342 | 114829 | 103783 | 94357 | 80417 | 94158 | 107851 | 103274 | 108582 | 116279 | 100950 | 81258 | 96616 | 85105 | 86182 | 69198 | 19884 | 17340 | 23470 | 16908 | 14799 | 12590 | 11360 | 13036 | 12149 | 9249 | 8997 |
|  | toluene-*d*_8_ | 15916 | 15305 | 16016 | 14900 | 14937 | 13317 | 11945 | 14543 | 13805 | 13382 | 24368 | 17571 | 16364 | 16545 | 16342 | 23729 | 17718 | 22109 | 22655 | 17274 | 20610 | 4278 | 4458 | 6061 | 2951 | 3760 | 6701 | 4148 | 6216 | 4853 | 4402 | 4746 |
| Group 2 | *n*-Buthyl acetate | 2541 | 3946 | 1886 | 2407 | 2320 | 2748 | 2183 | 2629 | 2296 | 2099 | 23377 | 16440 | 19206 | 23631 | 20021 | 24387 | 25441 | 27263 | 20997 | 37154 | 35512 | 17383 | 14897 | 16942 | 18179 | 16183 | 17085 | 19416 | 16104 | 16982 | 14011 | 12188 |
|  | Cyclohexanone | 465 | 783 | 303 | 426 | 417 | 447 | 312 | 430 | 347 | 307 | 5991 | 4110 | 4699 | 5987 | 4765 | 4702 | 4608 | 5211 | 4121 | 6550 | 6589 | 2302 | 1871 | 2172 | 2411 | 2002 | 1930 | 2149 | 1850 | 1903 | 1569 | 1309 |
|  | Ethyl acetate | 537 | 719 | 541 | 581 | 538 | 629 | 476 | 502 | 451 | 438 | 10373 | 8097 | 9486 | 8652 | 9609 | 10740 | 11492 | 10283 | 8839 | 13451 | 13479 | 8520 | 8516 | 8253 | 7971 | 9135 | 8701 | 9157 | 7225 | 8973 | 7051 | 7469 |
|  | Ethyl ether | 1400 | 2212 | 1210 | 1506 | 1508 | 1622 | 1203 | 1285 | 1274 | 967 | 30330 | 24017 | 27683 | 21462 | 29752 | 33296 | 34026 | 29126 | 27264 | 38035 | 35597 | 19507 | 23053 | 19742 | 18861 | 24441 | 23059 | 22975 | 18328 | 24464 | 16894 | 21275 |
|  | Ethylene glycol mono-*n*-butyl ether | 371 | 614 | 229 | 308 | 318 | 303 | 211 | 307 | 232 | 206 | 7246 | 5126 | 5160 | 7448 | 5609 | 1291 | 1974 | 2146 | 1884 | 2487 | 2853 | 2500 | 2239 | 2384 | 2448 | 2318 | 1859 | 2080 | 1927 | 1863 | 1485 | 1477 |
|  | Ethylene glycol monoethyl ether acetate | 99 | 139 | 73 | 89 | 92 | 74 | 55 | 62 | 57 | 53 | 1445 | 1090 | 1193 | 1482 | 1330 | 264 | 383 | 409 | 272 | 458 | 425 | 1098 | 985 | 1045 | 1072 | 1053 | 732 | 872 | 732 | 859 | 702 | 685 |
|  | *n*-Hexane | 51 | 73 | 56 | 55 | 58 | 69 | 56 | 59 | 55 | 41 | 2426 | 1900 | 2187 | 1956 | 2236 | 2574 | 2755 | 2570 | 2128 | 3490 | 3414 | 1006 | 1032 | 1018 | 977 | 1113 | 1130 | 1242 | 879 | 1209 | 907 | 910 |
|  | 4-Methylcyclohexanol | 544 | 897 | 346 | 486 | 491 | 512 | 350 | 490 | 373 | 336 | 7111 | 4961 | 5458 | 7081 | 5646 | 3251 | 4659 | 5441 | 3894 | 6937 | 6343 | 2570 | 2046 | 2404 | 2689 | 2180 | 2187 | 2405 | 2112 | 2136 | 1627 | 1429 |
|  | toluene-*d*_8_ | 1542 | 2287 | 1183 | 1486 | 1390 | 1583 | 1240 | 1515 | 1333 | 1202 | 21942 | 15011 | 17435 | 21961 | 19371 | 23431 | 23329 | 24767 | 19303 | 33686 | 31977 | 8932 | 7437 | 9341 | 9131 | 8220 | 14083 | 16211 | 12734 | 13740 | 11394 | 10061 |
| Group 3 | Cyclohexanol | 389572 | 347898 | 418227 | 258005 | 283752 | 467880 | 252503 | 313285 | 370179 | 195265 | 6541 | 10374 | 8478 | 9457 | 10966 | 5569 | 4133 | 4926 | 3985 | 5358 | 6643 | 2695 | 4996 | 3232 | 4343 | 3599 | 2912 | 3666 | 4080 | 2875 | 3106 | 2918 |
|  | Isopropyl acetate | 19780 | 17104 | 21550 | 14918 | 14324 | 23795 | 15323 | 18632 | 19852 | 13256 | 13053 | 17549 | 12142 | 15296 | 14629 | 10567 | 11432 | 11117 | 10918 | 13353 | 18533 | 5371 | 7029 | 6344 | 7240 | 6587 | 6063 | 7247 | 7659 | 6868 | 7157 | 5859 |
|  | Methyl acetate | 225845 | 206191 | 247719 | 191584 | 181810 | 218508 | 204599 | 178562 | 226532 | 134253 | 13464 | 11286 | 10972 | 10310 | 9668 | 9445 | 9108 | 7756 | 9294 | 10084 | 10675 | 7884 | 8694 | 8700 | 9846 | 8412 | 7462 | 7957 | 8544 | 7861 | 9018 | 7008 |
|  | Methyl ethyl ketone | 219780 | 208826 | 236280 | 178851 | 159573 | 256929 | 186179 | 182807 | 225438 | 132567 | 22227 | 24571 | 19588 | 22495 | 21986 | 15340 | 15709 | 15590 | 16155 | 19148 | 23734 | 12778 | 15396 | 14418 | 16535 | 14351 | 12845 | 14918 | 15750 | 14407 | 14927 | 12254 |
|  | Methyl isobutyl ketone | 86800 | 82405 | 97601 | 71807 | 69235 | 108132 | 77109 | 86616 | 102603 | 63153 | 2091 | 3406 | 2427 | 3124 | 3126 | 2017 | 2053 | 2087 | 1944 | 2383 | 3429 | 689 | 1206 | 853 | 1088 | 976 | 892 | 1102 | 1287 | 964 | 987 | 870 |
|  | *n*-Propyl acetate | 78932 | 72317 | 81715 | 60248 | 59163 | 97514 | 58889 | 70560 | 82809 | 38274 | 50926 | 73828 | 51801 | 68044 | 67046 | 45570 | 48585 | 47628 | 44808 | 54894 | 78973 | 33427 | 48488 | 40645 | 47939 | 43373 | 39622 | 48994 | 54848 | 43960 | 44448 | 38716 |
|  | 1,1,1-Trichloroethane | 2848984 | 2795051 | 3241373 | 2490617 | 2251124 | 3947374 | 2521919 | 2758136 | 3403689 | 1909291 | 187849 | 250593 | 172312 | 219095 | 215279 | 158025 | 169945 | 164885 | 160844 | 197165 | 279083 | 70346 | 96114 | 82975 | 99755 | 89935 | 82350 | 99250 | 107125 | 91037 | 92890 | 74842 |
|  | toluene-*d*_8_ | 13915 | 12841 | 15043 | 10481 | 10418 | 17584 | 11218 | 13491 | 15449 | 8760 | 17405 | 29711 | 21117 | 27456 | 29324 | 21229 | 20582 | 18649 | 17831 | 20886 | 30744 | 7200 | 11817 | 8645 | 10304 | 9391 | 13872 | 16885 | 19154 | 13740 | 14300 | 12354 |
| Group 4 | Benzene | 22203 | 20170 | 19064 | 23473 | 26921 | 22589 | 23566 | 19857 | 23002 | 23868 | 827 | 1468 | 1060 | 750 | 797 | 884 | 873 | 1060 | 1081 | 1225 | 929 | 652 | 389 | 296 | 300 | 443 | 449 | 385 | 385 | 458 | 422 | 381 |
|  | 1-Butanol | 47788 | 47487 | 43023 | 54182 | 59218 | 50935 | 50757 | 45191 | 51096 | 56281 | 5502 | 8415 | 6836 | 4282 | 5066 | 3551 | 3876 | 5302 | 4706 | 4825 | 4136 | 5213 | 3736 | 2922 | 3334 | 3910 | 3578 | 3176 | 3550 | 3682 | 3677 | 3152 |
|  | Chlorobenzene | 302863 | 244892 | 236415 | 291353 | 347261 | 288716 | 366309 | 261355 | 287710 | 332695 | 8853 | 16700 | 10088 | 7179 | 7623 | 7194 | 8943 | 11541 | 11115 | 13496 | 8958 | 7404 | 4731 | 3414 | 3984 | 5240 | 4949 | 4310 | 4166 | 5955 | 4339 | 4136 |
|  | Chloroform | 41192 | 40207 | 36378 | 44114 | 48614 | 45695 | 43925 | 39597 | 45108 | 44909 | 2347 | 3645 | 3029 | 1990 | 2217 | 2336 | 2442 | 3084 | 3019 | 3053 | 2684 | 1414 | 975 | 720 | 807 | 991 | 1211 | 875 | 987 | 1055 | 1037 | 908 |
|  | *o*-Cresol | 193377 | 169330 | 144119 | 137645 | 168479 | 57257 | 63966 | 46804 | 48793 | 48563 | 4571 | 5843 | 3518 | 3268 | 2857 | 106 | 141 | 192 | 199 | 167 | 110 | 2010 | 1648 | 1713 | 2092 | 1744 | 547 | 499 | 466 | 696 | 376 | 368 |
|  | *m*-Cresol | 222880 | 195568 | 167140 | 163390 | 196041 | 59816 | 66070 | 49208 | 52778 | 60683 | 5694 | 6934 | 4073 | 4161 | 3434 | 124 | 176 | 202 | 267 | 198 | 121 | 2226 | 1870 | 2042 | 2512 | 1975 | 371 | 359 | 330 | 508 | 257 | 250 |
|  | *p*-Cresol | 62555 | 55288 | 46662 | 45889 | 55134 | 12615 | 14095 | 10379 | 11205 | 12784 | 1567 | 1829 | 1157 | 1180 | 925 | 27 | - | - | 75 | 56 | 20 | 545 | 454 | 494 | 631 | 474 | 67 | 64 | 61 | 85 | 50 | 48 |
|  | *o*-Dichlorobenzene | 311593 | 254158 | 238084 | 245041 | 315616 | 256794 | 345590 | 242263 | 252634 | 307514 | 12197 | 19068 | 11196 | 8980 | 8791 | 6842 | 9751 | 12398 | 13368 | 14273 | 8583 | 12572 | 8460 | 6785 | 7883 | 9212 | 8575 | 7110 | 6399 | 10037 | 6656 | 6484 |
|  | 1,2-Dichloroethane | 15812 | 15348 | 14460 | 17982 | 20030 | 17825 | 17722 | 16005 | 18567 | 19901 | 1381 | 2281 | 1771 | 1117 | 1204 | 1289 | 1402 | 1840 | 1782 | 1745 | 1572 | 1194 | 805 | 583 | 644 | 808 | 869 | 724 | 785 | 835 | 806 | 708 |
|  | Dichloromethane | 180041 | 192539 | 159716 | 183403 | 208424 | 191809 | 172740 | 175331 | 177195 | 167306 | 9700 | 10937 | 11684 | 7619 | 8081 | 8866 | 9168 | 10685 | 11739 | 9564 | 9763 | 4488 | 3886 | 3271 | 3777 | 3890 | 3844 | 3352 | 4061 | 3837 | 4195 | 3476 |
|  | 1,2-Dichloropropane | 31736 | 27743 | 26740 | 33724 | 39962 | 33004 | 39239 | 30391 | 35987 | 41306 | 6259 | 12266 | 7388 | 5220 | 5485 | 5604 | 6425 | 8632 | 8081 | 10151 | 6839 | 2948 | 2001 | 1417 | 1600 | 2218 | 2261 | 1908 | 1832 | 2513 | 1917 | 1822 |
|  | *N,N*-Dimethylformamide | 81006 | 68888 | 66764 | 79947 | 91280 | 55203 | 62952 | 50498 | 57167 | 69803 | 3486 | 5197 | 3224 | 2997 | 2594 | 430 | 660 | 791 | 992 | 685 | 616 | 2485 | 1610 | 1616 | 1651 | 1831 | 580 | 521 | 496 | 692 | 448 | 467 |
|  | Isopenthyl alcohol | 426897 | 381130 | 350851 | 446538 | 490363 | 431667 | 441885 | 369269 | 435769 | 485213 | 13292 | 23490 | 15950 | 10865 | 11624 | 9275 | 10383 | 14183 | 13379 | 14804 | 10949 | 14498 | 9588 | 6906 | 8472 | 10043 | 9458 | 8485 | 8982 | 10231 | 9318 | 8348 |
|  | Isopentyl acetate | 751786 | 601893 | 590941 | 713169 | 847126 | 756144 | 965458 | 684489 | 796213 | 950900 | 21564 | 41436 | 25158 | 18377 | 19644 | 18591 | 23760 | 30088 | 29078 | 35268 | 22424 | 22577 | 15124 | 10420 | 13008 | 16490 | 16246 | 14692 | 14335 | 19859 | 14533 | 13827 |
|  | Methyl *n*-buthyl ketone | 11468 | 9644 | 8962 | 11608 | 13482 | 11334 | 13358 | 9891 | 10696 | 11382 | 350 | 640 | 420 | 296 | 321 | 333 | 359 | 455 | 444 | 561 | 359 | 160 | 93 | 66 | 85 | 101 | 96 | 90 | 92 | 122 | 88 | 93 |
|  | 4-Methylcyclohexanone | 278263 | 219591 | 217863 | 241521 | 294360 | 244005 | 329433 | 226563 | 234281 | 266703 | 7821 | 13066 | 7895 | 6190 | 6301 | 4731 | 6544 | 7965 | 8438 | 9258 | 5949 | 6214 | 4270 | 3163 | 3755 | 4606 | 4243 | 3933 | 3550 | 5373 | 3710 | 3512 |
|  | *n*-Pentyl acetate | 653804 | 509110 | 511356 | 600176 | 711126 | 635908 | 837892 | 576540 | 669891 | 809038 | 18650 | 35592 | 21795 | 16005 | 17126 | 15411 | 20671 | 24999 | 24744 | 29740 | 18590 | 19204 | 13108 | 9532 | 11492 | 14340 | 14020 | 12841 | 12148 | 17131 | 12449 | 11951 |
|  | Styrene | 717777 | 561886 | 557015 | 651400 | 788132 | 615114 | 829652 | 572810 | 616054 | 721418 | 20479 | 37553 | 22306 | 16255 | 17570 | 11681 | 16337 | 19642 | 20271 | 23287 | 14325 | 14056 | 9742 | 7272 | 8380 | 10710 | 9597 | 8448 | 8083 | 11622 | 8270 | 7867 |
|  | Tetrachloroethylene | 173413 | 143109 | 138204 | 173757 | 206958 | 174074 | 214507 | 158081 | 183504 | 210834 | 8549 | 17328 | 10183 | 7130 | 7592 | 7779 | 9259 | 12242 | 11534 | 14386 | 9188 | 11495 | 7391 | 5093 | 5985 | 8112 | 7863 | 6824 | 6697 | 9172 | 6960 | 6661 |
|  | Tetrachloromethane | 76224 | 69149 | 65939 | 81018 | 93058 | 79513 | 82932 | 70325 | 81254 | 84632 | 4983 | 8524 | 6073 | 4070 | 4387 | 4716 | 5113 | 6882 | 6434 | 7301 | 5458 | 3928 | 2667 | 1928 | 2081 | 2735 | 2929 | 2466 | 2583 | 2995 | 2772 | 2553 |
|  | Tetrahydrofuran | 104435 | 99017 | 91750 | 109612 | 126584 | 104681 | 105489 | 93599 | 105140 | 106562 | 4448 | 7358 | 5842 | 3685 | 4224 | 4222 | 4307 | 5731 | 5470 | 5685 | 4753 | 5156 | 3629 | 2778 | 2944 | 3770 | 3901 | 3319 | 3548 | 3898 | 3782 | 3429 |
|  | Toluene | 487905 | 409568 | 390283 | 491850 | 575531 | 481554 | 576225 | 432688 | 493879 | 551571 | 20029 | 40392 | 24476 | 17156 | 18242 | 18773 | 21688 | 29075 | 26796 | 33462 | 22467 | 10371 | 6755 | 4791 | 5554 | 7403 | 7483 | 6369 | 6210 | 8380 | 6454 | 6256 |
|  | Trichloroethylene | 55292 | 49187 | 46775 | 59095 | 67507 | 58399 | 62061 | 51635 | 58974 | 61183 | 2184 | 4168 | 2780 | 1899 | 2025 | 2204 | 2303 | 3200 | 3019 | 3663 | 2557 | 2921 | 1835 | 1303 | 1454 | 1930 | 2085 | 1747 | 1782 | 2178 | 1892 | 1771 |
|  | *o*-Xylene | 1003400 | 780258 | 780492 | 922029 | 1110796 | 929207 | 1239749 | 853695 | 923706 | 1082057 | 33543 | 65128 | 37623 | 27174 | 29335 | 26441 | 34901 | 44309 | 43043 | 52677 | 32616 | 21938 | 14749 | 10037 | 12456 | 16275 | 15094 | 13423 | 12769 | 18867 | 13237 | 12894 |
|  | *m*-Xylene | 1003040 | 785023 | 780947 | 928044 | 1092225 | 941096 | 1238289 | 868473 | 916338 | 1066465 | 31287 | 62207 | 35554 | 25439 | 27655 | 26041 | 34067 | 43288 | 41650 | 51167 | 32146 | 24696 | 16438 | 11375 | 13892 | 27307 | 17273 | 15236 | 14595 | 21378 | 15288 | 14757 |
|  | *p*-Xylene | 554312 | 434836 | 429344 | 514474 | 615296 | 520974 | 690964 | 479855 | 513616 | 598598 | 15782 | 31631 | 18257 | 12982 | 14234 | 13304 | 17313 | 21853 | 21098 | 26082 | 16398 | 12307 | 8387 | 5949 | 7058 | 27203 | 8860 | 7810 | 7422 | 10772 | 7773 | 7443 |
|  | toluene-*d*_8_ | 11223 | 9305 | 8570 | 11195 | 12926 | 10787 | 13005 | 9558 | 10717 | 11683 | 20530 | 38316 | 23233 | 16210 | 17198 | 17754 | 20466 | 27222 | 26342 | 31547 | 21148 | 11504 | 7556 | 5367 | 6137 | 8027 | 13625 | 11649 | 12172 | 15374 | 11992 | 11562 |

Table S3 Raw data (peak areas) for 3 commercial active samplers in concentration of 2 × administrative levels.

| 2 × Guide line values | | SIBATA | | | | | | | | | | SKC | | | | | | | | | | | GASTEC | | | | | | | | | | |
| --- | --- | --- | --- | --- | --- | --- | --- | --- | --- | --- | --- | --- | --- | --- | --- | --- | --- | --- | --- | --- | --- | --- | --- | --- | --- | --- | --- | --- | --- | --- | --- | --- | --- |
|  |  | Standard | | | | | Sample | | | | | Standard | | | | | Sample | | | | | | Standard | | | | | Sample | | | | | |
|  |  | No. 1 | No. 2 | No. 3 | No. 4 | No. 5 | No. 1 | No. 2 | No. 3 | No. 4 | No. 5 | No. 1 | No. 2 | No. 3 | No. 4 | No. 5 | No. 1 | No. 2 | No. 3 | No. 4 | No. 5 | No. 6 | No. 1 | No. 2 | No. 3 | No. 4 | No. 5 | No. 1 | No. 2 | No. 3 | No. 4 | No. 5 | No. 6 |
| Group 1 | Acetone | 458748 | 454474 | 481596 | 468276 | 440852 | 447830 | 337103 | 353968 | 339510 | 321004 | 384895 | 439484 | 438559 | 332461 | 303062 | 218004 | 199327 | 196166 | 144933 | 171868 | 130863 | 173202 | 185936 | 170141 | 165173 | 153457 | 119030 | 190835 | 125678 | 147061 | 170667 | 147072 |
|  | *cis*-1,2-Dichloroethylene | 860088 | 788005 | 872960 | 865354 | 812246 | 841491 | 727097 | 785900 | 767641 | 673354 | 142618 | 161273 | 146263 | 150729 | 155680 | 116430 | 115895 | 120731 | 105808 | 113225 | 91886 | 73763 | 83408 | 77593 | 72217 | 66247 | 57876 | 107838 | 66455 | 83804 | 90540 | 79402 |
|  | 1,4-Dioxane | 121516 | 117397 | 130618 | 142206 | 131784 | 140770 | 106855 | 119822 | 116701 | 108588 | 11103 | 13781 | 10270 | 12710 | 13599 | 8517 | 8626 | 9547 | 8733 | 8106 | 7132 | 5608 | 6045 | 5737 | 5468 | 5361 | 4287 | 7726 | 4812 | 6230 | 6518 | 5871 |
|  | toluene-*d*_8_ | 54889 | 60317 | 58263 | 65472 | 64590 | 61441 | 47384 | 54075 | 48701 | 44414 | 35376 | 46071 | 30651 | 41047 | 43641 | 24181 | 26278 | 27785 | 27196 | 22844 | 20906 | 29793 | 31547 | 31978 | 30207 | 30409 | 25751 | 46772 | 30162 | 38054 | 41092 | 37494 |
| Group 2 | 2-Butanol | 120596 | 101306 | 81751 | 129696 | 103215 | 104985 | 104635 | 95965 | 76607 | 57019 | 43783 | 31521 | 38777 | 30904 | 40578 | 29625 | 31733 | 30730 | 25509 | 26751 | 30343 | 38663 | 49810 | 40468 | 17079 | 46672 | 41409 | 34347 | 27472 | 26152 | 40738 | 42134 |
|  | Ethylene glycol monoethyl ether acetate | 26420 | 21982 | 14987 | 30610 | 21892 | 23799 | 25583 | 25830 | 16450 | 12877 | 6462 | 3625 | 5333 | 3954 | 6853 | 3391 | 5417 | 5393 | 4425 | 3402 | 3476 | 3354 | 4306 | 3738 | 1327 | 4085 | 3567 | 3362 | 2517 | 2112 | 3343 | 3663 |
|  | Isobutyl acetate | 648739 | 468647 | 373056 | 655272 | 543643 | 585906 | 650373 | 644521 | 421424 | 325560 | 290675 | 154606 | 225604 | 157616 | 269207 | 169210 | 227598 | 226756 | 200136 | 168298 | 174931 | 217579 | 281847 | 227887 | 77745 | 264724 | 255107 | 217828 | 170688 | 144291 | 237165 | 259345 |
|  | Isobutyl alcohol | 2464 | 1925 | 1296 | 1961 | 1969 | 2035 | 1961 | 1830 | 1463 | 1244 | 18279 | 16304 | 19542 | 16185 | 21132 | 14777 | 16066 | 15014 | 13123 | 13605 | 15492 | 10234 | 13084 | 10608 | 4580 | 11933 | 10449 | 8883 | 6830 | 6381 | 9930 | 10483 |
|  | Isopropyl alcohol | 69003 | 48244 | 30908 | 52357 | 62259 | 55712 | 54488 | 43765 | 41818 | 29475 | 284686 | 241359 | 281924 | 273863 | 289397 | 227946 | 207260 | 218419 | 142555 | 188338 | 232120 | 177053 | 232106 | 206277 | 107077 | 208227 | 170799 | 146435 | 117399 | 123278 | 175451 | 172962 |
|  | toluene-*d*_8_ | 50595 | 36448 | 36652 | 60571 | 45016 | 47155 | 53863 | 53855 | 35303 | 27533 | 38787 | 21189 | 31030 | 22869 | 37472 | 22620 | 30938 | 31173 | 28016 | 23597 | 24528 | 34686 | 43701 | 35590 | 12765 | 38482 | 37478 | 31040 | 25011 | 20402 | 32509 | 34859 |
| Group 3 | Cyclohexanone | 234911 | 154597 | 303109 | 354053 | 331684 | 212790 | 256593 | 310819 | 189111 | 162695 | 16375 | 18810 | 12471 | 17029 | 16864 | 10254 | 13895 | 10724 | 10801 | 5630 | 10566 | 8256 | 9591 | 10552 | 8314 | 8685 | 6429 | 6635 | 4766 | 5911 | 7210 | 5920 |
|  | Ethyl ether | 540855 | 381651 | 404346 | 614599 | 669075 | 349697 | 538536 | 487935 | 421830 | 353110 | 130799 | 146734 | 91744 | 133942 | 104810 | 75919 | 102930 | 88406 | 76254 | 36195 | 94592 | 123044 | 132729 | 142337 | 105533 | 107026 | 97135 | 97440 | 61243 | 78698 | 105125 | 65698 |
|  | Ethylene glycol monoethyl ether | 15561 | 11280 | 15770 | 17030 | 15847 | 9252 | 11485 | 13426 | 9022 | 8425 | 3698 | 4534 | 3133 | 3954 | 4038 | 258 | 500 | 479 | 296 | 269 | 457 | 3640 | 3977 | 4494 | 3576 | 3462 | 2003 | 2043 | 1422 | 1875 | 2269 | 1639 |
|  | *n*-Hexane | 71037 | 43554 | 57931 | 76147 | 75135 | 52381 | 66890 | 69890 | 52486 | 44268 | 6825 | 8746 | 5572 | 7125 | 7490 | 5216 | 8148 | 6099 | 5935 | 2935 | 5944 | 5006 | 5561 | 5246 | 4782 | 4653 | 4158 | 4386 | 2782 | 3643 | 4361 | 3291 |
|  | toluene-*d*_8_ | 35183 | 24062 | 38630 | 46009 | 44354 | 34045 | 35299 | 42863 | 30962 | 23713 | 25049 | 27893 | 20758 | 25009 | 28772 | 20168 | 29677 | 22097 | 23893 | 23221 | 20127 | 25850 | 30033 | 27193 | 25825 | 25604 | 22132 | 23516 | 15860 | 20472 | 24507 | 19376 |
| Group 4 | *n*-Buthyl acetate | 193305 | 165063 | 285837 | 196239 | 303852 | 322547 | 236127 | 188991 | 263259 | 223038 | 67630 | 71702 | 85144 | 59198 | 55244 | 55534 | 71241 | 79987 | 75702 | 50489 | 59329 | 19021 | 17414 | 16170 | 21537 | 14762 | 14593 | 15595 | 16696 | 17950 | 20387 | 14664 |
|  | Ethyl acetate | 168102 | 152235 | 174390 | 162266 | 179188 | 169306 | 162047 | 139286 | 181324 | 167255 | 32089 | 28516 | 35656 | 26269 | 28511 | 27662 | 27502 | 32260 | 29337 | 25469 | 25722 | 9503 | 8806 | 9603 | 11156 | 7722 | 7602 | 8356 | 8780 | 8457 | 9638 | 8835 |
|  | Ethylene glycol mono-*n*-butyl ether | 116408 | 93952 | 187400 | 99435 | 168978 | 165530 | 121898 | 90439 | 120264 | 115072 | 16125 | 18133 | 20322 | 15431 | 14801 | 5271 | 6161 | 7215 | 6363 | 4482 | 5353 | 2512 | 2480 | 2568 | 2868 | 2242 | 1934 | 2163 | 1727 | 2060 | 2147 | 2147 |
|  | 4-Methylcyclohexanol | 162092 | 126267 | 261727 | 130451 | 230223 | 234976 | 168862 | 121293 | 160283 | 160239 | 17225 | 19426 | 21069 | 16777 | 15047 | 12401 | 13875 | 16628 | 15633 | 10652 | 13181 | 2694 | 2517 | 2347 | 3166 | 2266 | 1947 | 2123 | 1970 | 2147 | 2461 | 2014 |
|  | toluene-*d*_8_ | 20833 | 16242 | 24372 | 17816 | 26239 | 27516 | 21056 | 17471 | 24148 | 21280 | 25340 | 25795 | 29836 | 22090 | 20113 | 20049 | 25171 | 28392 | 27301 | 19073 | 21536 | 7745 | 6925 | 6263 | 8361 | 5597 | 5569 | 5847 | 6531 | 6678 | 7572 | 5347 |
| Group 5 | Cyclohexanol | 282656 | 265128 | 278133 | 448811 | 280212 | 322248 | 316854 | 348204 | 306081 | 263607 | 26623 | 25753 | 22615 | 19256 | 30526 | 14319 | 13956 | 16385 | 38632 | 19431 | 12156 | 3529 | 3426 | 3736 | 3063 | 2739 | 2549 | 3173 | 2801 | 3085 | 2966 | 2844 |
|  | Methyl isobutyl ketone | 61076 | 57710 | 56142 | 77596 | 58481 | 64090 | 66420 | 71603 | 71194 | 60168 | 7545 | 6666 | 6091 | 4927 | 8382 | 5188 | 4907 | 5662 | 12610 | 6698 | 3757 | 1006 | 911 | 907 | 813 | 607 | 819 | 735 | 800 | 851 | 831 | 852 |
|  | *n*-Propyl acetate | 364785 | 349446 | 342042 | 485203 | 344037 | 379065 | 406745 | 447731 | 410748 | 344050 | 181744 | 157580 | 142448 | 126968 | 227682 | 138789 | 117029 | 150733 | 333608 | 176345 | 95908 | 44086 | 39860 | 40578 | 36584 | 28881 | 39762 | 34862 | 37313 | 38839 | 38169 | 39094 |
|  | 1,1,1-Trichloroethane | 3129790 | 2914333 | 2920210 | 3371948 | 2905698 | 3144106 | 3192874 | 3319141 | 3467010 | 3171240 | 613710 | 520552 | 488135 | 462029 | 798557 | 534701 | 456233 | 548688 | 1080593 | 633684 | 363387 | 99737 | 87813 | 89755 | 80203 | 65286 | 83873 | 76885 | 88018 | 81457 | 79878 | 81495 |
|  | toluene-*d*_8_ | 24720 | 23560 | 23133 | 32962 | 23513 | 27291 | 28321 | 30520 | 28673 | 24480 | 26895 | 24651 | 20845 | 16936 | 29102 | 18383 | 17253 | 20517 | 49923 | 25564 | 13131 | 6760 | 6078 | 6093 | 5540 | 4407 | 5192 | 4861 | 5061 | 5306 | 5047 | 5089 |
| Group 6 | Isopropyl acetate | 121054 | 105589 | 107778 | 120722 | 125961 | 128637 | 113578 | 117218 | 93736 | 113094 | 34662 | 46641 | 35040 | 33403 | 38840 | 41805 | 40753 | 37667 | 39119 | 40770 | 50340 | 11638 | 10685 | 10543 | 10501 | 10032 | 8322 | 10528 | 9164 | 9721 | 9772 | 9622 |
|  | Methyl acetate | 153994 | 160534 | 166136 | 181089 | 177504 | 164250 | 138746 | 158821 | 137988 | 136195 | 30330 | 30683 | 27626 | 25400 | 28090 | 28705 | 30130 | 24230 | 25131 | 27257 | 33644 | 7640 | 7651 | 8019 | 7162 | 7993 | 6020 | 8366 | 5906 | 6549 | 6637 | 6079 |
|  | Methyl ethyl ketone | 30180 | 31772 | 32926 | 39020 | 41699 | 37606 | 33805 | 36260 | 32239 | 32422 | 44758 | 54381 | 43231 | 39505 | 47733 | 48228 | 50466 | 42659 | 44195 | 47468 | 57196 | 14060 | 13629 | 13932 | 13196 | 13019 | 10175 | 13028 | 10414 | 11147 | 10953 | 10456 |
|  | toluene-*d*_8_ | 25439 | 16280 | 19859 | 20712 | 21902 | 25003 | 22519 | 19744 | 18696 | 20193 | 12048 | 22203 | 14905 | 13246 | 19369 | 19527 | 18517 | 17785 | 19939 | 20770 | 22992 | 5848 | 5114 | 4747 | 4957 | 5077 | 3854 | 4816 | 4735 | 4378 | 4390 | 4249 |
| Group 7 | Isopenthyl alcohol | 281338 | 243351 | 272934 | 244528 | 252609 | 191652 | 218980 | 191780 | 227951 | 271858 | 26168 | 31088 | 30943 | 33344 | 34361 | 26202 | 26037 | 22676 | 28803 | 29554 | 40782 | 8792 | 8427 | 8680 | 9680 | 8577 | 6428 | 7638 | 10766 | 8068 | 7659 | 12937 |
|  | Isopentyl acetate | 543106 | 420164 | 515047 | 404957 | 401951 | 326075 | 417552 | 344346 | 454250 | 602120 | 37207 | 50431 | 54209 | 58898 | 56668 | 51481 | 58875 | 46120 | 70670 | 66494 | 94830 | 12726 | 13188 | 13839 | 15347 | 12907 | 12375 | 12519 | 21801 | 13802 | 12955 | 22485 |
|  | *n*-Pentyl acetate | 475712 | 355141 | 452732 | 353878 | 333674 | 271514 | 353650 | 285080 | 389258 | 420974 | 30972 | 43018 | 45858 | 50479 | 47773 | 40305 | 50688 | 38838 | 59283 | 54372 | 78015 | 10692 | 11280 | 12336 | 13044 | 11317 | 11171 | 10354 | 18469 | 11619 | 11132 | 18348 |
|  | Tetrahydrofuran | 76665 | 70647 | 75504 | 68650 | 65410 | 43133 | 56018 | 57147 | 68408 | 71208 | 9875 | 11287 | 10728 | 11533 | 14905 | 12479 | 10728 | 10659 | 11500 | 13124 | 17269 | 3648 | 3197 | 3352 | 3688 | 3373 | 2692 | 2996 | 3983 | 3083 | 3233 | 5050 |
|  | toluene-*d*_8_ | 19270 | 16021 | 17633 | 13094 | 13447 | 10893 | 13691 | 12174 | 15136 | 19280 | 13194 | 17703 | 18396 | 20391 | 19470 | 20240 | 19293 | 16238 | 24200 | 24568 | 32860 | 4285 | 4249 | 4269 | 4878 | 4056 | 3686 | 3959 | 6555 | 4430 | 4255 | 7229 |
| Group 8 | Benzene | 17292 | 15908 | 14686 | 15439 | 17262 | 18909 | 18252 | 17508 | 15558 | 17830 | 1632 | 2431 | 1977 | 3301 | 1970 | 2106 | 2324 | 1912 | 1820 | 1863 | 2389 | 332 | 480 | 321 | 484 | 466 | 345 | 581 | 396 | 508 | 400 | 421 |
|  | 1-Butanol | 18654 | 17666 | 16887 | 19413 | 22968 | 21958 | 23403 | 23255 | 19624 | 22961 | 10174 | 13931 | 11411 | 18287 | 12657 | 9468 | 10909 | 8127 | 7881 | 8015 | 9456 | 3075 | 4236 | 3360 | 4902 | 4441 | 3008 | 4772 | 3612 | 4118 | 3486 | 3873 |
|  | Chlorobenzene | 281506 | 243872 | 200775 | 201666 | 243846 | 266046 | 271192 | 247669 | 217140 | 230723 | 14674 | 20436 | 18501 | 28870 | 16109 | 19628 | 20922 | 12086 | 17962 | 14291 | 19472 | 3944 | 5439 | 3347 | 5949 | 5216 | 3679 | 6130 | 3873 | 5431 | 4134 | 4680 |
|  | Chloroform | 38365 | 34974 | 33598 | 34543 | 38035 | 40924 | 40108 | 39973 | 34239 | 40223 | 4925 | 8951 | 7664 | 12221 | 7750 | 8042 | 8909 | 6542 | 7046 | 6886 | 8341 | 814 | 1168 | 820 | 1307 | 1192 | 842 | 1398 | 1058 | 1179 | 986 | 1057 |
|  | *o*-Cresol | 119740 | 103405 | 94565 | 75033 | 92270 | 27298 | 36664 | 31590 | 29060 | 29483 | 6535 | 8176 | 7994 | 9774 | 7396 | 665 | 781 | 377 | 633 | 423 | 547 | 1540 | 1730 | 1379 | 1963 | 2116 | 367 | 716 | 468 | 600 | 543 | 431 |
|  | *m*-Cresol | 148759 | 129958 | 117786 | 95063 | 114787 | 28476 | 37705 | 32341 | 30814 | 31169 | 7749 | 10376 | 9756 | 11602 | 9456 | 768 | 799 | 429 | 694 | 559 | 640 | 1757 | 2004 | 1688 | 2275 | 2476 | 276 | 491 | 337 | 454 | 390 | 327 |
|  | *p*-Cresol | 38447 | 33752 | 30978 | 24784 | 29436 | 6029 | 7914 | 6873 | 6483 | 6560 | 2133 | 2762 | 2742 | 3216 | 2518 | 122 | 127 | 96 | 115 | - | 117 | 412 | 497 | 418 | 584 | 624 | 53 | 84 | 65 | 83 | 74 | 63 |
|  | *o*-Dichlorobenzene | 261793 | 237539 | 215368 | 170784 | 218170 | 202395 | 253262 | 229761 | 210838 | 197931 | 19324 | 25231 | 24374 | 33523 | 22361 | 24172 | 24558 | 14634 | 21663 | 15703 | 20950 | 7227 | 9520 | 6584 | 10529 | 10117 | 6131 | 10656 | 6330 | 8479 | 7493 | 7319 |
|  | 1,2-Dichloroethane | 10216 | 9903 | 9596 | 10458 | 12079 | 12908 | 13158 | 13358 | 11610 | 13734 | 2799 | 3846 | 3172 | 5162 | 3361 | 3308 | 3886 | 2855 | 2815 | 2757 | 3527 | 647 | 942 | 704 | 1086 | 1027 | 719 | 1170 | 858 | 974 | 805 | 885 |
|  | Dichloromethane | 169996 | 165593 | 167736 | 153573 | 168837 | 166277 | 170261 | 187963 | 159422 | 193011 | 19416 | 24848 | 21293 | 29642 | 24569 | 22058 | 24009 | 22463 | 18037 | 18949 | 19553 | 3261 | 4058 | 3649 | 5030 | 4418 | 3396 | 4686 | 4451 | 3923 | 3816 | 3854 |
|  | 1,2-Dichloropropane | 19160 | 18236 | 15943 | 17626 | 21032 | 23399 | 23699 | 22270 | 19468 | 22286 | 10370 | 15602 | 13592 | 22363 | 11810 | 14590 | 15982 | 9645 | 13209 | 11413 | 15306 | 1685 | 2474 | 1505 | 2517 | 2420 | 1684 | 3004 | 1906 | 2585 | 1963 | 2177 |
|  | *N,N*-Dimethylformamide | 57395 | 49584 | 43821 | 42974 | 52711 | 36571 | 40728 | 36948 | 29768 | 35062 | 5648 | 8303 | 7228 | 10380 | 7339 | 2227 | 2738 | 1215 | 1832 | 1517 | 1951 | 1394 | 1924 | 1599 | 2109 | 2009 | 646 | 1022 | 676 | 993 | 777 | 748 |
|  | Methyl *n*-buthyl ketone | 8961 | 7768 | 6462 | 6951 | 8111 | 8771 | 9002 | 8349 | 6785 | 7979 | 634 | 960 | 786 | 1214 | 724 | 749 | 913 | 564 | 710 | 703 | 784 | 83 | 120 | 71 | 119 | 111 | 79 | 133 | 86 | 118 | 87 | 101 |
|  | 4-Methylcyclohexanone | 207171 | 183356 | 165452 | 134735 | 173862 | 172870 | 198447 | 178590 | 161662 | 159330 | 12225 | 17273 | 15965 | 21913 | 14551 | 14918 | 15728 | 9327 | 13133 | 10010 | 12951 | 3534 | 4822 | 2943 | 5290 | 4694 | 3014 | 5312 | 3383 | 4796 | 3629 | 3976 |
|  | Styrene | 562093 | 494097 | 421902 | 383622 | 476381 | 472997 | 509567 | 458578 | 414762 | 420385 | 33749 | 47008 | 43672 | 65359 | 37353 | 39073 | 42522 | 24434 | 36147 | 28062 | 38137 | 7986 | 10910 | 6717 | 11753 | 10655 | 6818 | 11726 | 7415 | 10377 | 8037 | 9064 |
|  | Tetrachloroethylene | 207338 | 186413 | 152376 | 163122 | 195614 | 221711 | 217826 | 203721 | 174243 | 198442 | 15754 | 22346 | 19485 | 32208 | 16579 | 20758 | 23433 | 13474 | 19588 | 16279 | 22598 | 6531 | 9133 | 5218 | 9320 | 8562 | 5981 | 10491 | 6632 | 9065 | 6934 | 7756 |
|  | Tetrachloromethane | 108035 | 95767 | 87696 | 90829 | 100920 | 113487 | 108466 | 103893 | 92072 | 106498 | 9460 | 12979 | 10567 | 17334 | 10385 | 11582 | 13158 | 9385 | 9885 | 9698 | 12309 | 2312 | 3132 | 2113 | 3367 | 3188 | 2362 | 3880 | 2689 | 3334 | 2715 | 2849 |
|  | Toluene | 399214 | 352229 | 289879 | 310780 | 364017 | 413352 | 404928 | 375627 | 322957 | 362261 | 37417 | 51894 | 45454 | 74342 | 40036 | 48616 | 54672 | 32198 | 44497 | 38385 | 51834 | 5902 | 8305 | 5081 | 8365 | 8002 | 5724 | 9958 | 6362 | 8664 | 6713 | 7389 |
|  | Trichloroethylene | 62692 | 55560 | 49582 | 53881 | 61184 | 70607 | 68248 | 66144 | 57185 | 66373 | 3851 | 5712 | 4759 | 8030 | 4683 | 5539 | 6217 | 3948 | 4859 | 4554 | 5850 | 1575 | 2195 | 1377 | 2241 | 2127 | 1574 | 2716 | 1816 | 2294 | 1803 | 1959 |
|  | *o*-Xylene | 892866 | 785142 | 661379 | 610551 | 764037 | 798325 | 856239 | 772111 | 689138 | 714469 | 53425 | 75248 | 69273 | 106116 | 57899 | 72078 | 78655 | 44967 | 66282 | 52162 | 72279 | 12271 | 17277 | 10139 | 18132 | 16267 | 11516 | 19571 | 12206 | 17540 | 13163 | 15084 |
|  | *m*-Xylene | 879090 | 732330 | 613367 | 581147 | 722412 | 792403 | 836740 | 763854 | 668780 | 705164 | 52155 | 74445 | 67653 | 105396 | 55928 | 71205 | 79810 | 44565 | 66364 | 52911 | 72902 | 13517 | 18845 | 11177 | 19090 | 17669 | 12751 | 21601 | 13614 | 19100 | 14444 | 16851 |
|  | *p*-Xylene | 451598 | 399030 | 335152 | 312211 | 390986 | 416524 | 442212 | 400085 | 356184 | 372996 | 26395 | 37153 | 33800 | 52848 | 28421 | 35786 | 39716 | 22369 | 33693 | 26157 | 36815 | 6816 | 9493 | 5663 | 9596 | 8904 | 6263 | 10928 | 6886 | 9656 | 7354 | 8476 |
|  | toluene-*d*_8_ | 21960 | 18861 | 15619 | 16802 | 19907 | 22408 | 22080 | 20375 | 17511 | 19843 | 15419 | 21234 | 18079 | 30843 | 17146 | 20129 | 21776 | 13010 | 17725 | 15240 | 20368 | 4235 | 5959 | 3653 | 6041 | 5739 | 4135 | 7178 | 4638 | 6142 | 4793 | 5257 |

a)
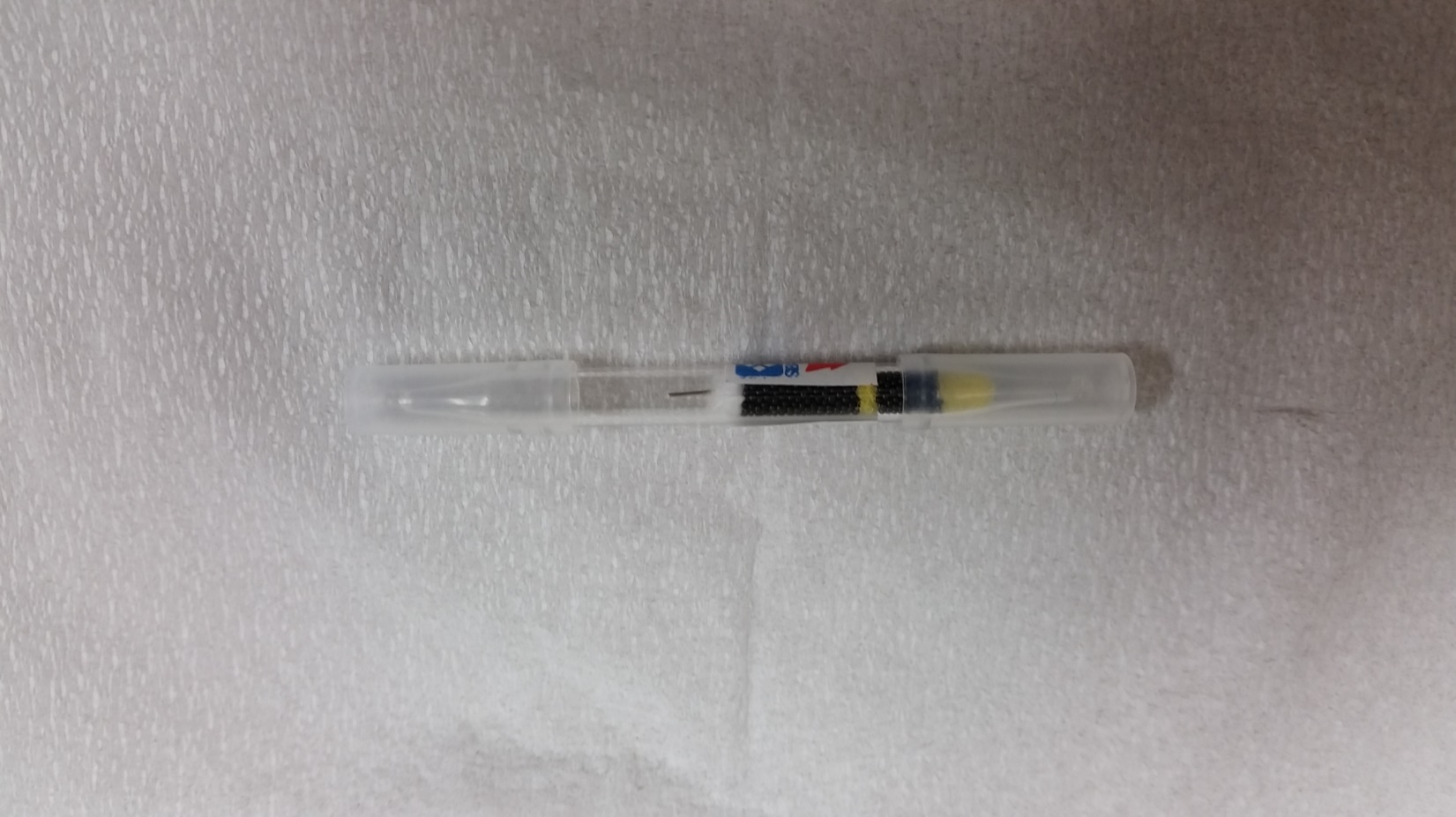


b)
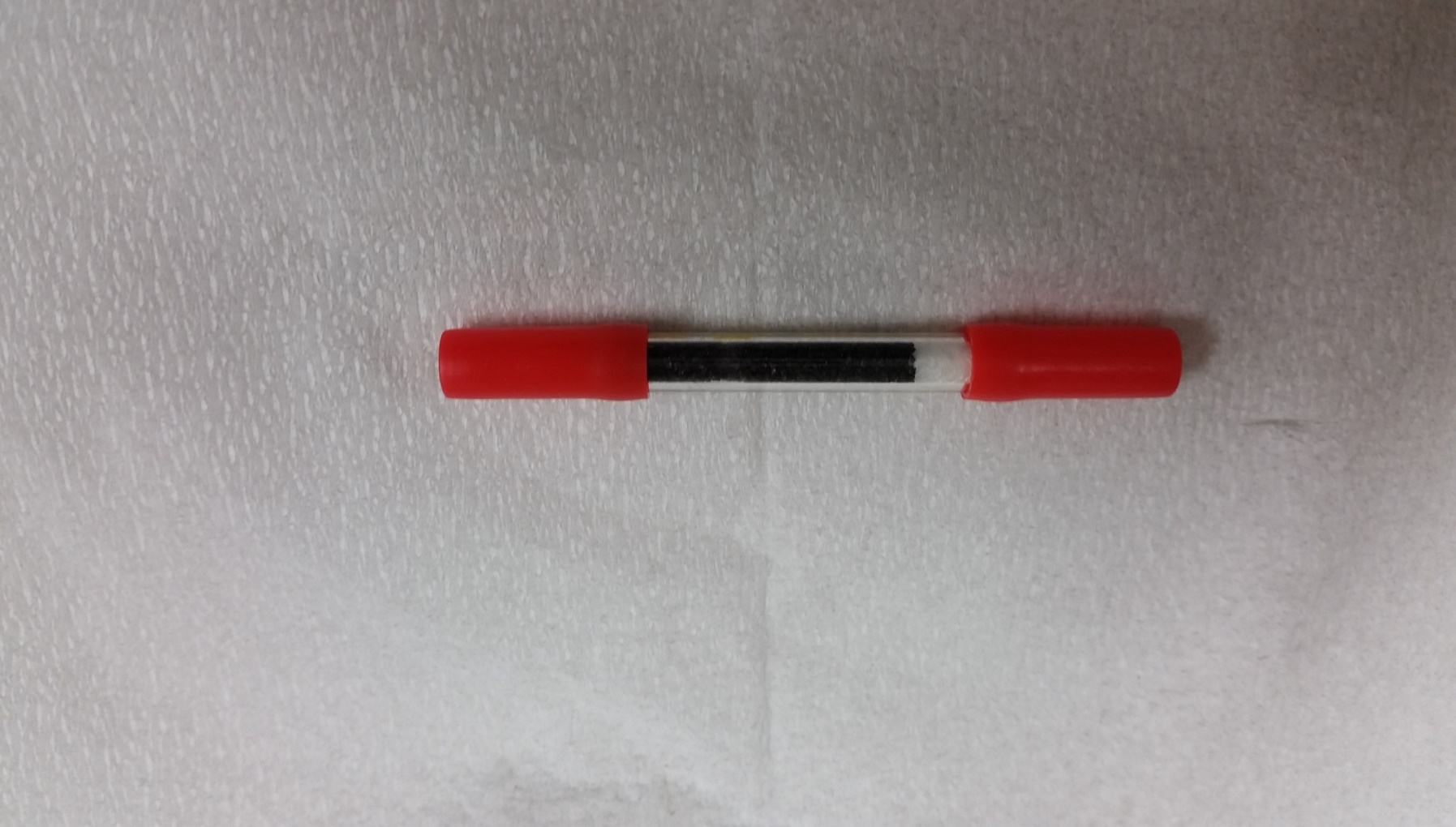


c)
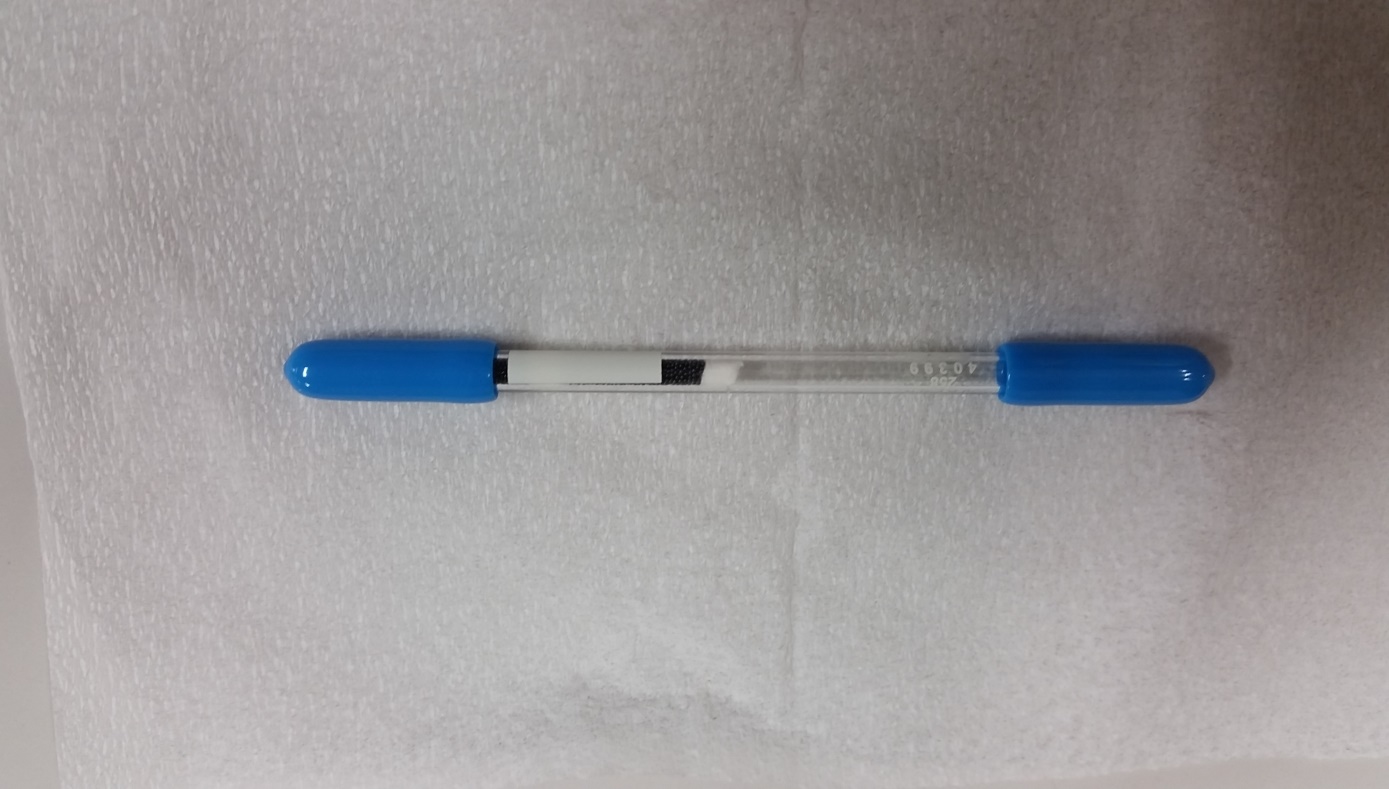


Fig. S1 Pictures of 3 commercial active samplers (a: SIBATA; b: SKC; c: GASTEC).

Fig. S2 Recovery rates for 3 commercial active samplers (a: SIBATA; b: SKC; c: GASTEC) in different concentrations

(0.5 × administrative levels, 1 × administrative levels, and 2 × administrative levels).
